# Knowledge graph visualization and retrospective analysis of genetic research on pediatric cardiomyopathy (2000–2024)

**DOI:** 10.3389/fcvm.2026.1780649

**Published:** 2026-06-02

**Authors:** Xiaohua Han, Feng Wang, Guifang Wang

**Affiliations:** 1 Department of Pediatrics, Xinxiang Central Hospital, The Fourth Clinical College of Xinxiang Medical University, Xinxiang, Henan, China; 2 Department of Cardiology, Children’s Hospital of Fudan University, Shanghai, China

**Keywords:** genetics, pediatric cardiomyopathy, precision medicine, quantitative analysis, topic modeling

## Abstract

**Background:**

Pediatric cardiomyopathy is a leading cause of heart failure and sudden cardiac death in children, posing a severe threat to their health and lives while imposing a heavy burden on families and society. It has become a significant public health challenge. The aim of this retrospective study is to systematically review global research articles on pediatric cardiomyopathy genetics, revealing its knowledge structure and evolutionary trajectory.

**Methods:**

Bibliometric methods and natural language processing techniques were jointly applied to analyze research articles on pediatric cardiomyopathy genetics from the Web of Science Core Collection (WOSCC) and PubMed databases. CiteSpace software was utilized to construct national collaboration networks and co-occurrence/evolution maps of keywords, while BERTopic modeling was employed for topic modeling of article abstracts. The macro-structure and micro-semantics of pediatric cardiomyopathy genetics research were systematically investigated, and finally a multi-dimensional knowledge map was constructed.

**Results:**

Over the past 25 years, research teams from 71 countries and regions have published 1,438 articles, demonstrating fluctuating growth in publishing activity. The United States, China, and the United Kingdom are core publishing nations, with the U.S. occupying a central position in publication volume, total citations, and international collaboration networks. This study identified five core themes in pediatric cardiomyopathy genetics, including diverse disease classification and diagnostic/therapeutic mechanisms, systematically revealing the field's research trajectory toward intelligent and precision-oriented transformation. Based on keyword timeline analysis and emergence analysis, the research evolution progressed through three phases: single-gene screening to genomic sequencing (early 2000s–2010), multi-omics integration (early 2010s–2020), and precision medicine with dynamic monitoring (early 2020s–2024).

**Conclusion:**

This study further demonstrates that genetic research in pediatric cardiomyopathy is increasingly integrated into digital healthcare, rapidly advancing toward intelligent and precise diagnosis and treatment. The integration of multi-omics data and artificial intelligence supports personalized risk assessment, dynamic monitoring, and early warning, thereby driving the transformation toward data-driven pediatric cardiovascular health management.

## 1 Introduction

In recent years, cardiac-related sudden death has dominated all sudden death cases, accounting for a staggering proportion exceeding 90%. This poses a significant psychological impact and threat to public and social stability. Among children, a special population, cardiomyopathy has been identified as the primary cause of cardiac-related sudden death. This grim reality highlights that improving early screening, genetic counseling, and targeted management for pediatric cardiomyopathy is a key priority in pediatric medicine (1, 2). It is also an urgent public health issue related to population health and the reduction of family and social burdens.

Pediatric cardiomyopathy refers to a group of cardiac diseases caused by structural or functional abnormalities of the myocardium, clinically manifested as inappropriate myocardial hypertrophy or cardiac chamber dilation (3). Its incidence ranges from 1.13/100,000 to 1.24/100,000, lower than congenital heart disease but a common cause of pediatric heart failure and sudden cardiac death. Approximately 1/3 of affected children ultimately die or require heart transplantation (4–6). Since the discovery of the first cardiomyopathy-causing gene in 1990, genetic research has progressively deepened, and genetic testing technology has evolved from basic exploration to clinical practice (7, 8). With the application of karyotyping, chromosomal microarray analysis, gene sequencing, whole-exome sequencing, and multi-omics technologies (9–11), genetic testing powered by artificial intelligence and big data platforms has become a vital tool for cardiomyopathy diagnosis, precise classification, and risk assessment. This advancement is driving the transformation of clinical practice toward digital and personalized approaches (12, 13).

This study combined bibliometric analysis and natural language processing. Based on 2000–2024 publications from Web of Science Core Collection (WOSCC) and PubMed, we used CiteSpace for knowledge network analysis and BERTopic for in-depth semantic mining. Addressing the current research gap in systematic knowledge graph construction and dynamic evolution pathway analysis, this study aims to systematically identify core research themes, track hotspot evolution trajectories, and reveal knowledge structure correlations through large-scale articles analysis, thereby providing empirical evidence and pathway references for the development and clinical translation of genetic research in pediatric cardiomyopathy.

## 2 Data and methodology

### 2.1 Data sources and retrieval strategies

**Figure 1:**
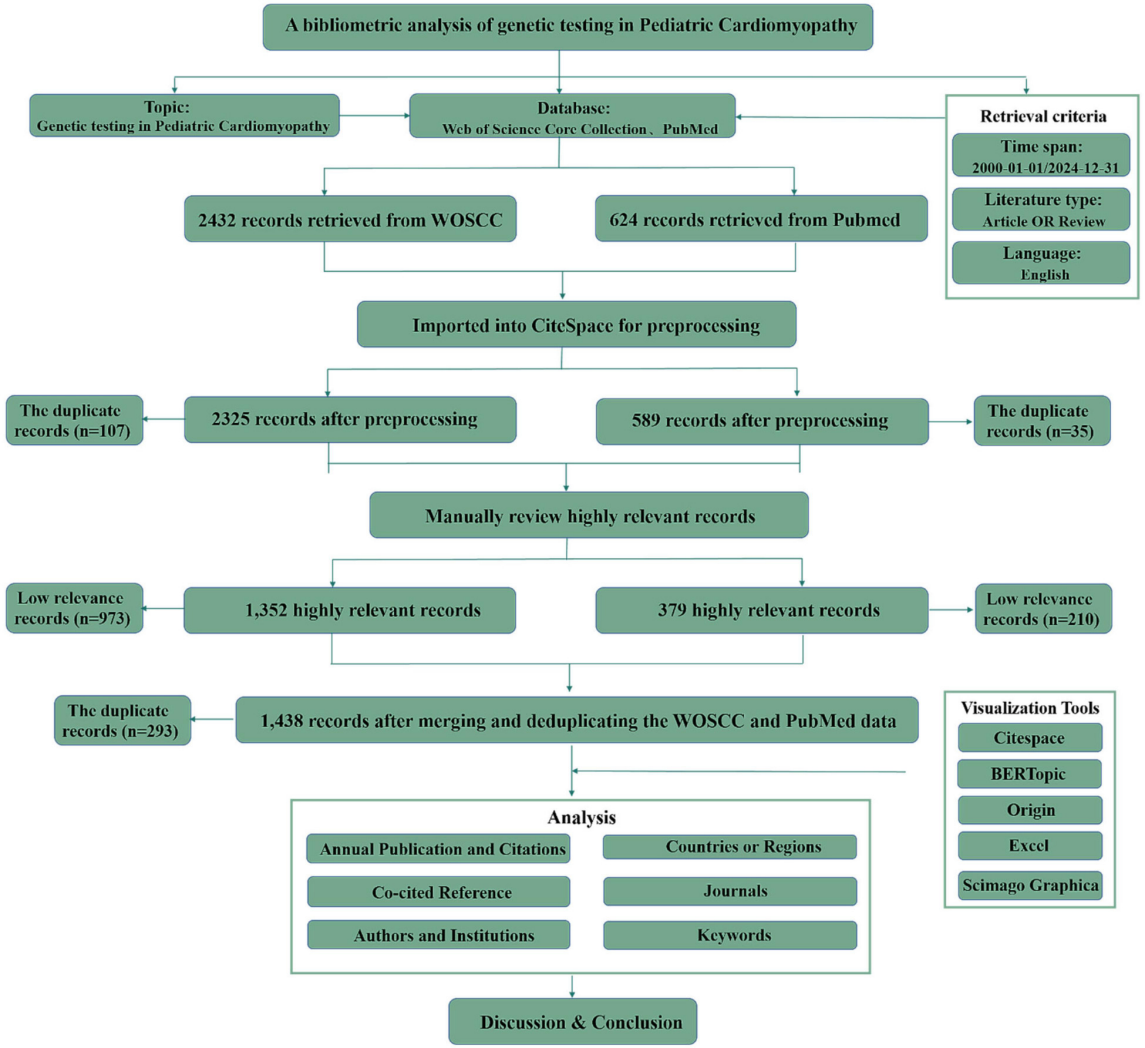
The work flow diagram of the study.

To ensure the comprehensiveness of article retrieval and the reliability of the results, this study adopted a cross-database search and validation strategy. The same search query was used to simultaneously retrieve the WOSCC and PubMed databases. All article retrieval and data collection were completed on September 8, 2025, after which the complete bibliographic records were downloaded as the dataset. The research flowchart is shown in Figure 1. The search period was set from January 1, 2000, to December 31, 2024, with the language restricted to “English.” The WOSCC subject search query was: TS = (cardiomyopathy) AND TS = (gene OR genotype OR genetic OR mutation OR variant) AND TS = (neonate OR infant OR child OR pediatric OR adolescent), yielding *n* = 2,536 records. After filtering for “article” and “review article” types, the number of records was reduced to *n* = 2,432. The PubMed subject search query was ((“cardiomyopathies”[Mesh] AND (gene[tiab] OR genotype[tiab] OR genetic[tiab] OR mutation[tiab] OR variant[tiab])) AND (neonatal[tiab] OR infant[tiab] OR child[tiab] OR pediatric[tiab] OR adolescent[tiab])), resulting in *n* = 1,625 records. Applying the same exclusion and inclusion criteria, the final number of records obtained was *n* = 624.

Then, the 2,432 records retrieved from the WOSCC database and the 624 records retrieved from PubMed were imported separately into CiteSpace 6.3.R1 software for data preprocessing, including format standardization, duplicate identification, and removal. After processing, 107 duplicates were removed from the WOSCC database and 35 from PubMed, resulting in a total of 142 duplicates removed. Consequently, 2,325 valid records remained from WOSCC and 589 from PubMed.

To further ensure that the included records were highly relevant to the research topic on the genetics of pediatric cardiomyopathy, two researchers independently screened the titles and abstracts of the deduplicated records based on pre-established inclusion and exclusion criteria, and consulted the full text when necessary. In case of disagreement, a third researcher was consulted for arbitration. After rigorous screening, 973 records from WOSCC and 210 from PubMed were excluded for not meeting the topic requirements, yielding a total of 1,183 excluded records. Finally, 1,352 WOSCC records and 379 PubMed records directly relevant to this manuscript were obtained.

To ensure the comprehensiveness of research data and the reliability of conclusions, this study conducted cross-database validation. First, the data from WOSCC and PubMed were merged and standardized. Manual verification identified 293 duplicate records across the two databases. After removing duplicates, 1,438 valid records were obtained for this manuscript.

To systematically assess the reliability and complementarity of multi-database retrieval, this study evaluated the cross-database complementarity between WOSCC and PubMed based on three dimensions: records overlap, topical coverage, and quality characteristics. The quantitative results are shown in Table 1. The low overlap proportion (Jaccard=0.20) indicates that duplication is generally low, with each database containing unique content, and that a single database cannot fully cover all relevant articles in this research field. In terms of topical coverage, WOSCC focuses more on bibliometric characteristics, multidisciplinary research, and highly cited outputs, whereas PubMed covers a broader range of biomedical outputs, including regional and applied research. The two databases exhibit clear complementarity in research topics. Regarding quality characteristics, both databases primarily include peer-reviewed journal articles, ensuring the academic rigor of the included studies. Comprehensive evaluation shows that combining WOSCC and PubMed for cross-database retrieval and validation can effectively reduce the indexing bias associated with a single database, improve the completeness and representativeness of the dataset, and ensure robust and reliable subsequent analyses.

**Table 1:** Quantitative results of cross-database validation between WOSCC and pubMed.

Indicator	Value	Indicator	Value
Total records in WoSCC	1,352	Proportion of records unique to WOSCC	78.3%
Total records in PubMed	379	Proportion of records unique to Pubmed	22.7%
Overlapping records	293	Marginal complementarity rate of PubMed to WOS	6.4%
Union	1,438	Marginal complementarity rate of WOS to PubMed	279%
Jaccard index	0.204		

### 2.2 Inclusion and exclusion criteria

Inclusion criteria: (1) study subjects are children or adolescents ≤18 years of age; (2) the research involves genetic analysis of cardiomyopathy, including but not limited to: genetic mutation screening, genotype-phenotype association, genetic testing methods (e.g., Sanger sequencing, whole-exome sequencing), genetic counseling, and inheritance patterns of familial cardiomyopathy.

Exclusion criteria: (1) studies exclusively involving genetic research on adult cardiomyopathy (>18 years of age); (2) studies focusing solely on non-genetic cardiomyopathy (e.g., viral myocarditis, drug/chemotherapy-induced cardiomyopathy, Kawasaki disease-associated cardiomyopathy); (3) genetics is mentioned only as background but is not the core research focus (e.g., studies primarily discussing imaging, pharmacotherapy, or surgical procedures); (4) article types such as conference abstracts, editorials, letters, case reports (unless they involve systematic analysis of multiple cases), guidelines, or popular science articles; (5) full text cannot be obtained and the abstract provides insufficient information to determine relevance; (6) duplicate publications (when the same author or team publishes multiple articles on the same study, only the study of higher quality is included); (7) articles that have been explicitly retracted by the journal or publisher.

Resolution of disagreements: any disagreements between the two researchers during the article screening process were resolved by involving a third reviewer, who made the final decision. This reviewer strictly adhered to the “direct relevance” principle, requiring that a study must simultaneously meet the following three criteria for inclusion: (1) the study sample or analysis population explicitly includes children with cardiomyopathy; (2) the core research topic involves genetics, including but not limited to the detection rate of genetic mutations, associations between specific genes and phenotypes, and the clinical utility of genetic testing; (3) the results section reports quantitative or qualitative data related to genetics. If genetic content appears only in the introduction or discussion sections, with no substantive genetic analysis provided in the methods or results sections, the study is considered not relevant to the subject of this manuscript.

### 2.3 Research methods

To better reflect the research themes and evolutionary trajectory of pediatric cardiomyopathy genetics, the CiteSpace visualization tool and BERTopic topic modeling method were combined for bibliometric analysis. On the one hand, CiteSpace was employed to systematically organize the quantitative characteristics of current genetic analysis research on pediatric cardiomyopathy, exploring research hotspots and developmental trends in the field. On the other hand, to delve deeper into the semantic content and latent thematic structures of the articles, this study utilized the BERTopic method to extract meaningful information from the abstracts of the included publications for thematic mining. Through clustering, semantically consistent thematic clusters were identified, enabling the categorization of research topics and further identification of emerging trends and future research directions. CiteSpace emphasizes the revelation of macro-level structures and evolutionary patterns, while BERTopic focuses on the extraction of micro-level semantics and thematic connotations. Through cross-validation and result integration, this study aims to construct a multidimensional knowledge map, providing a robust methodological and evidentiary foundation for subsequent discussions and conclusions.

## 3 Results

### 3.1 Temporal analysis of publication

**Figure 2:**
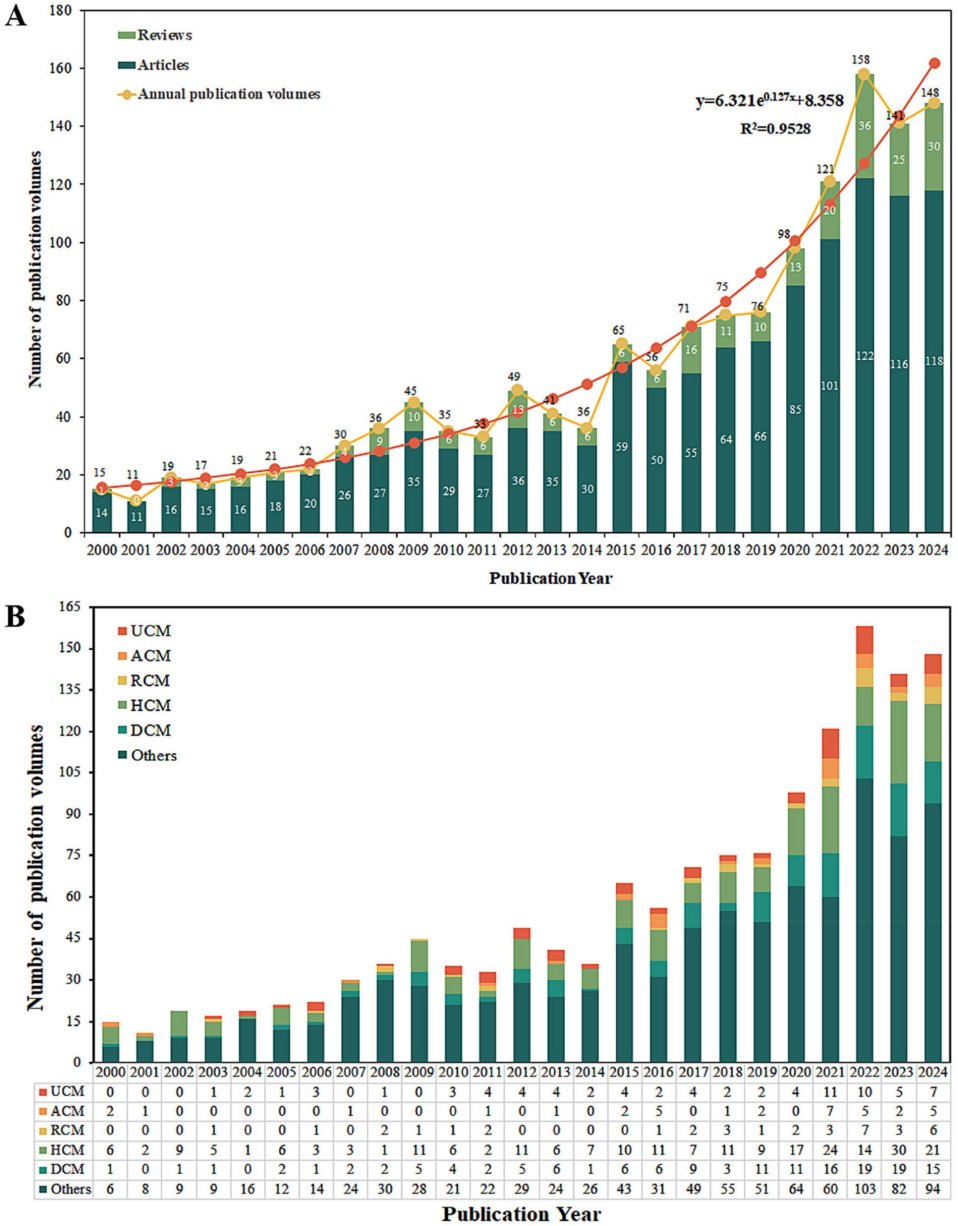
Publication volume of genetic studies on pediatric cardiomyopathies. **(A)** Annual publication volume. **(B)** Annual publication volume distribution for clinical phenotypes-genes.

This study utilized Excel software to create the annual distribution chart of publications on pediatric cardiomyopathy genetics (Figure 2). Since 2000, the annual number of publications in this field has exhibited an overall fluctuating upward trend, with the lowest point occurring in 2001 at only 11 articles and peaking in 2022 at 158 articles. Origin software was used to perform exponential curve fitting on the annual publication volume (Figure 2A), yielding a fitted curve with an R2 value of 0.9528. Here, a higher R2 value indicates better fitting quality (14). The fitted curve reflects a rapid upward trend in research intensity and development of pediatric cardiomyopathy genetics, with no inflection point observed. This indicates that the field will remain a hot research topic for a considerable period in the future.

Based on the quantitative changes in annual publications, research on genetic cardiomyopathy in children can be roughly divided into three stages. The period from 2000 to 2006 was the initial phase, with fewer than 30 publications per year, showing slow growth, indicating a preliminary exploratory stage of research. The phase from 2007 to 2018 marked a growth period, with publications exhibiting a wave-like upward trend, reflecting gradually increasing research activity and heightened academic attention. The stage from 2019 to 2024 represents an explosive growth period, where the number of publications surged rapidly, peaking in 2022, demonstrating that the field has entered a phase of rapid development. Overall, the publication volume exhibits a fluctuating growth trend, with fluctuations generally aligning with the annual publication counts. Additionally, original articles constitute over 80% of the total publications.

Hypertrophic cardiomyopathy (HCM) and dilated cardiomyopathy (DCM) remain research focal points due to their high clinical incidence, consistently exceeding 60% and showing an upward trend (Figure 2B). Conversely, research on relatively rare uremic cardiomyopathy (UCM), arrhythmogenic cardiomyopathy (ACM), and restrictive cardiomyopathy (RCM)-has shown a wave-like increase in published articles. This indicates growing academic attention toward understanding and studying these subtypes, thereby filling existing gaps in knowledge. Moreover, approximately 40%–85% of publications were categorized as “Others” due to unclear clinical phenotype descriptions or descriptions of more than one clinical phenotype. This suggests that future research should further strengthen the standardization of phenotype descriptions.

### 3.2 Country distribution and collaboration analysis

The absolute number of articles published by a country in a specific discipline reflects its research activity, technological innovation capacity, and collaborative engagement with other nations (15).

#### 3.2.1 Analysis of country distribution

**Figure 3:**
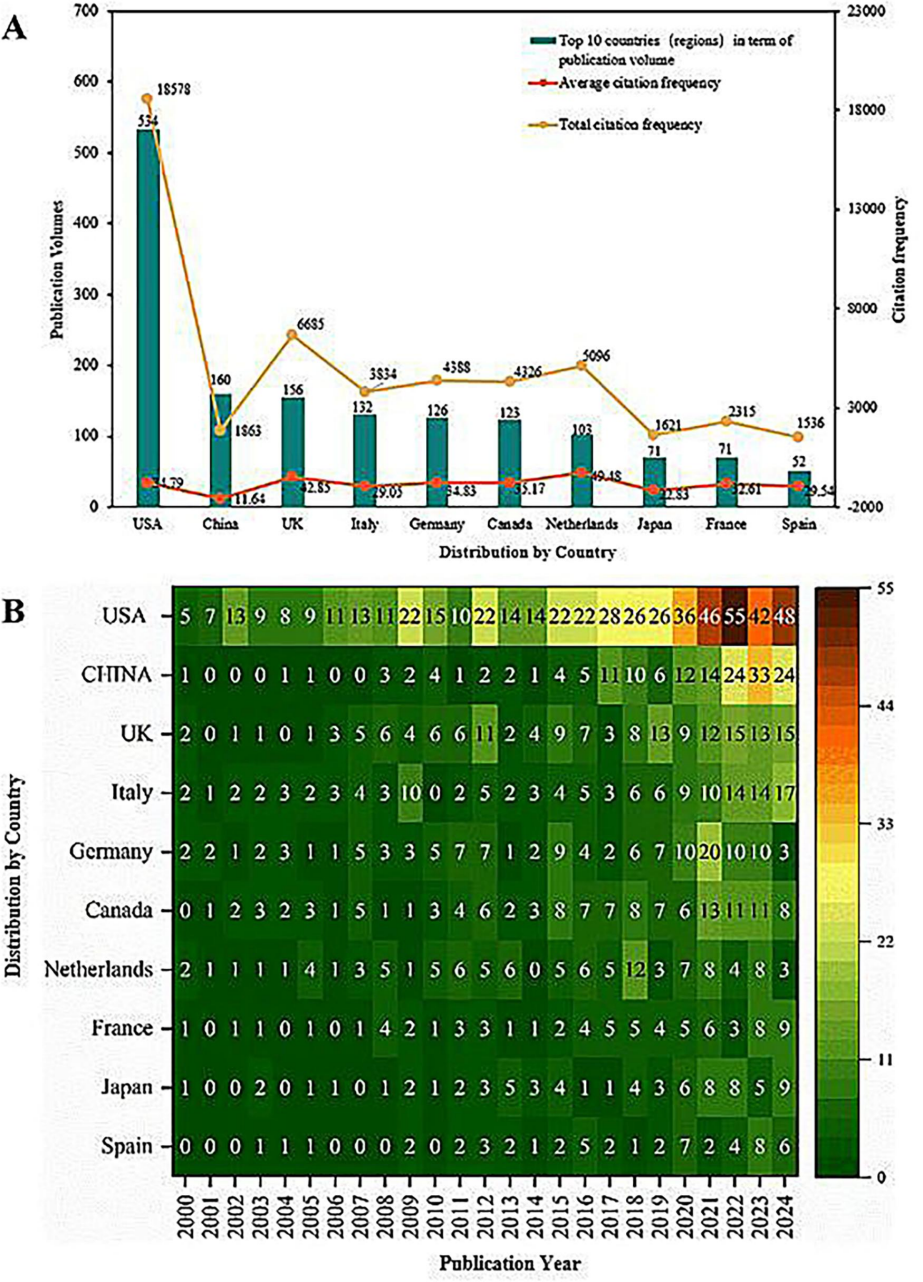
Analysis of the top 10 countries in terms of publication volume. **(A)** Publication volume, total citations, and average citations. **(B)** Heatmap of annual publications.

A statistical analysis of 1,438 publications revealed that 71 countries have participated in pediatric cardiomyopathy genetics research over the past 25 years. By examining the total citations, average citations per article, total publications, and annual publications of the top 10 countries in terms of publication count (Figure 3), it was found that the top 3 countries were the United States, China, and the United Kingdom. The top 5 countries accounted for approximately 77.05% of the total publications, indicating a high geographical concentration. In terms of academic influence, the United States, the United Kingdom, and the Netherlands led in total citations, while the Netherlands, the United Kingdom, and Canada stood out in average citations per article, reflecting the high quality and recognition of their research outcomes.

The heat map of annual publication counts for the top 10 countries (Figure 3B) shows a general upward trend in publications over time, with particularly heightened research activity in recent years. The United States has maintained a dominant position throughout the entire research period. China has seen a significant increase in publications since 2010, emerging as a major contributor. Countries such as the United Kingdom, Italy, and Germany have also demonstrated high research activity during specific years.

#### 3.2.2 Analysis of international collaboration

**Figure 4:**
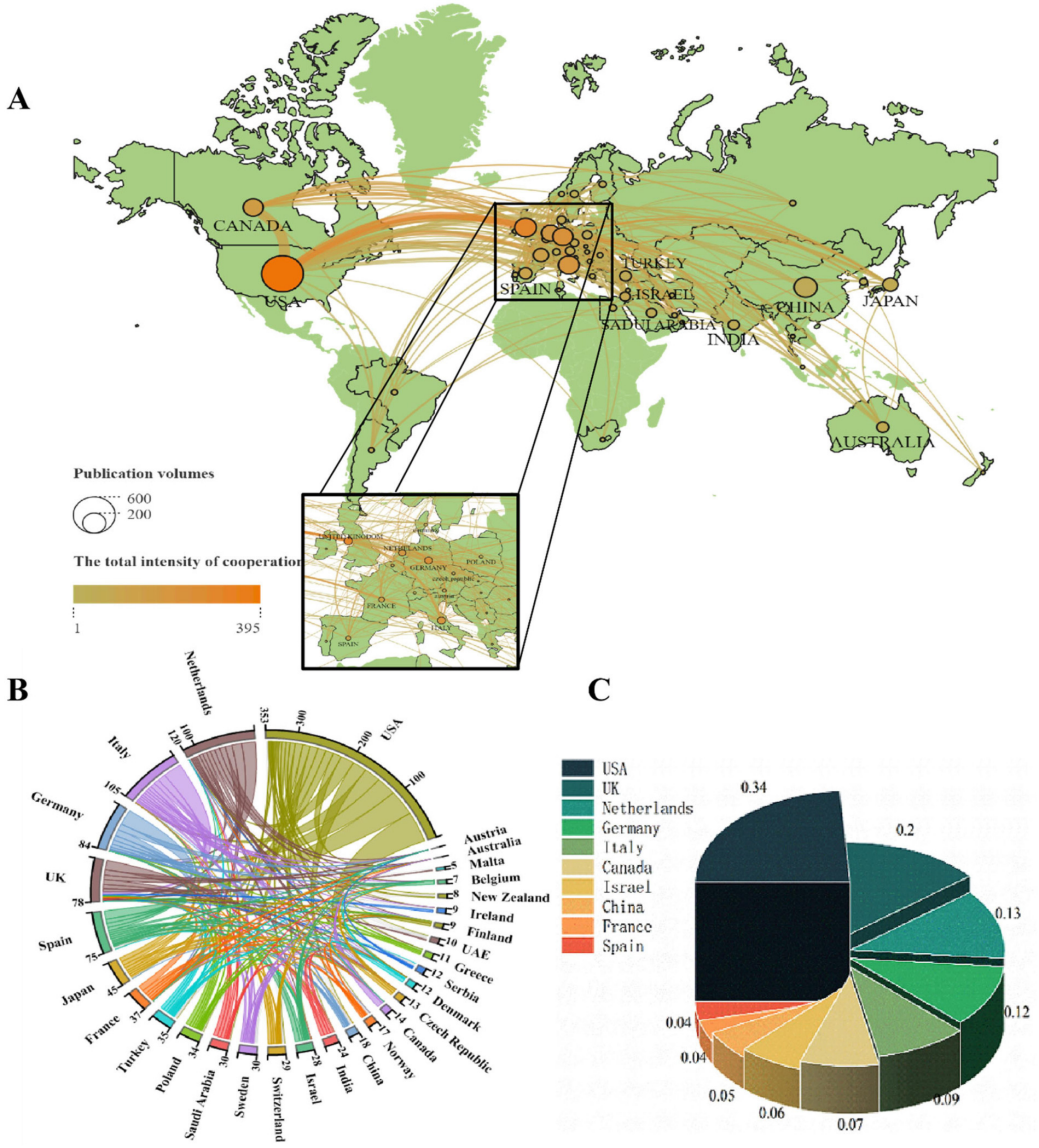
Analysis of country collaboration. **(A)** National collaboration network geographic display. **(B)** Chord diagram of national collaboration relationships. **(C)** The top 10 countries in terms of betweenness centrality.

SCImago Graphica and Origin software were used to create national collaboration network maps, analyzing cooperative relationships and academic exchanges between countries (16). Figures 4A,B display the geographical distribution and collaboration network of the 71 publishing countries, respectively. The network exhibits a radial collaboration pattern centered around the United States, indicating its pivotal role in the global scientific research collaboration network. The betweenness centrality results in Figure 4C further confirm the conclusion, with the United States having the highest betweenness centrality (0.34). This suggests that the U.S. holds significant influence in international research collaborations, with its institutions and scholars likely playing key roles in multiple international projects. Additionally, England, Italy, and Germany rank among the top five in both publication volume and centrality, indicating these three countries produce substantial research outcomes, maintain close international connections, and sustain consistent international influence. The regional collaboration clusters have formed among European countries (e.g., UK-Italy, Germany-Netherlands), demonstrating notable geographical and group characteristics.

### 3.3 Analysis of institutional collaboration network

**Figure 5:**
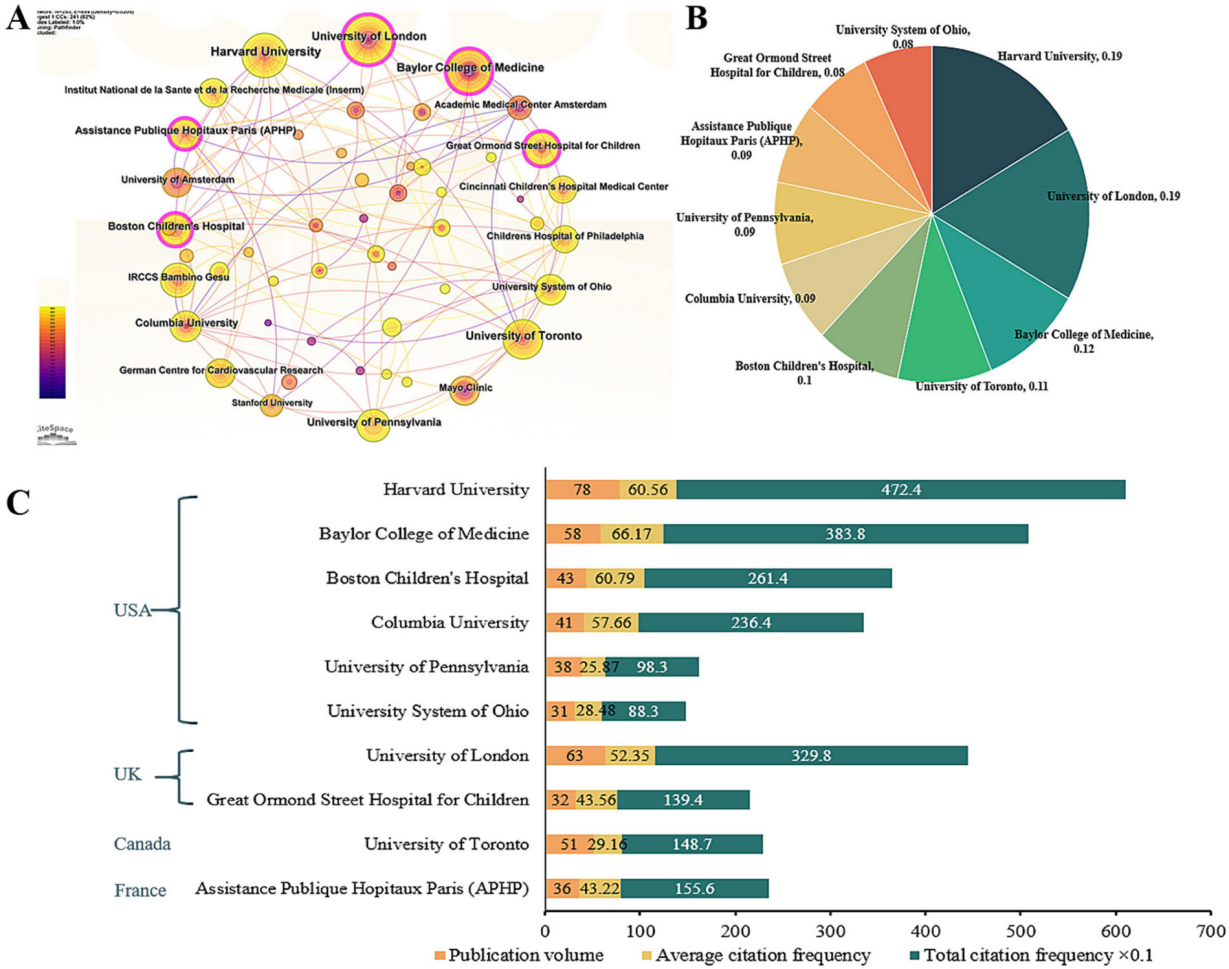
Analysis of institutional collaboration network. **(A)** Institutional collaboration network map. **(B)** The top 10 institutions in terms of betweenness centrality. **(C)** Publication volume, total citations, and average article citations for the top 10 institutions.

Figure 5 visually illustrates the collaborative relationships among institutions. Figure 5A highlights 19 research institutions that have published over 25 articles. This network diagram aids in identifying key research institutions, as well as the distribution of scientific capabilities and knowledge flow among institutions (17). The network reveals extensive collaborative ties among research institutions, with dense connections indicating frequent exchanges and cooperation. The data in Figures 5B,C show that 6 out of the top 10 institutions by publication volume are from the United States, indicating a relatively concentrated research strength in the U.S., with universities playing a leading role. Institutions ranked in the top 5 by betweenness centrality, such as Baylor College of Medicine, Assistance Publique-Hôpitaux de Paris (AP-HP), and Great Ormond Street Hospital for Children, play pivotal roles in this research domain, serving as key nodes for knowledge flow and collaboration. Overall, the institutional collaboration network is characterized by U.S. universities taking the lead, with participation from multiple countries and institutions, driving continuous advancement and development in genetic research on pediatric cardiomyopathy.

Furthermore, as seen in Figure 5C, among the top 10 institutions by publication volume, those with higher total citation counts tend to be the ones with more publications. This indicates that high-output institutions are more likely to gain academic attention and recognition. In particular, United States-based institutions like Harvard University and Baylor College of Medicine exhibit significantly higher total citation counts than others, further underscoring America's research strength and influence.

### 3.4 Keyword analysis

#### 3.4.1 Keyword timeline and emergence analysis

**Figure 6:**
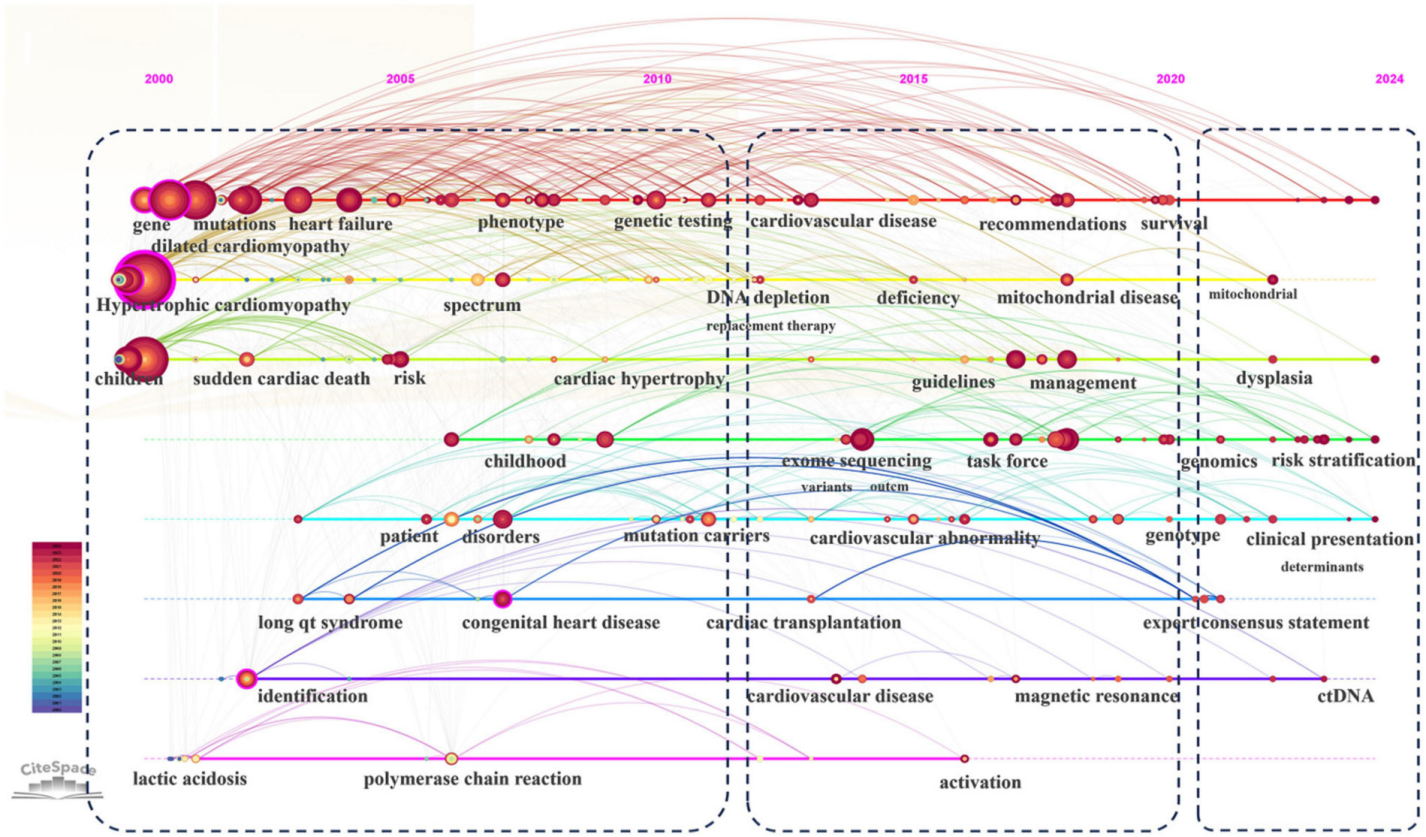
The co-occurrence network timeline map of keywords.

**Figure 7:**
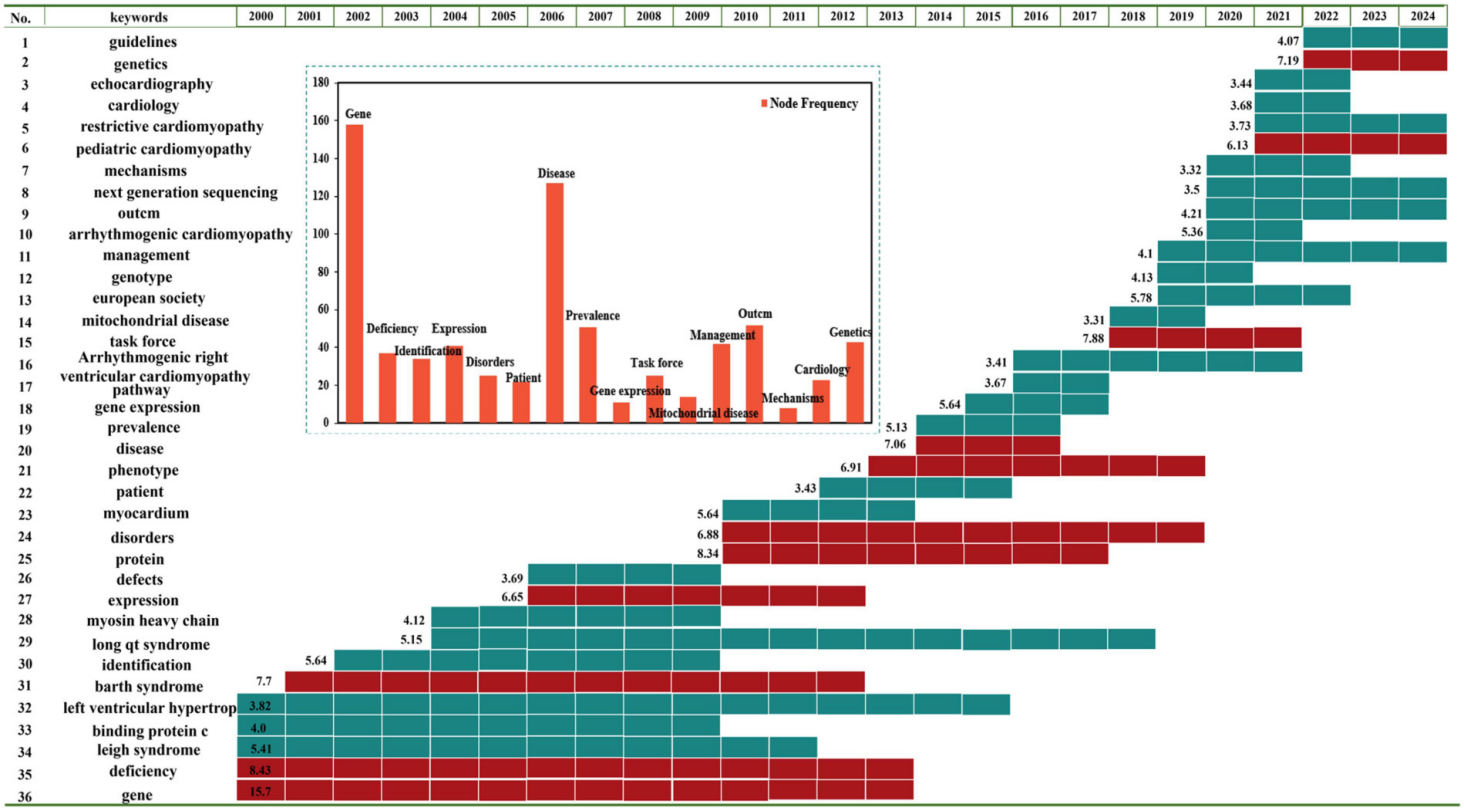
Burst map of keywords.

To comprehensively track the migration trends of research hotspots, CiteSpace software was used to generate the co-occurrence network timeline map of keywords (Figure 6) and conduct burst analysis (Figure 7). The timeline map clearly reveals the phased migration of research themes alongside technological evolution, which can be divided into three main stages:

1. Single-gene targeted screening phase (early 2000s–2010)

This phase primarily relied on Sanger sequencing technology to conduct targeted testing for individual known pathogenic genes (e.g., *MYH7* and *MYBPC3* in hypertrophic cardiomyopathy). In clinical applications for pediatric cardiomyopathy, it was mainly used for confirming familial cardiomyopathy cases (18). However, due to technological limitations, the detection rate of disease-causing genes during this phase was low, making it difficult to cover rare genes or *de novo*mutations. It also struggled to address the genetic heterogeneity of cardiomyopathy and had limited diagnostic efficacy for pediatric metabolic cardiomyopathies (e.g., mitochondrial diseases).

2. High-Throughput Sequencing and Multi-Omics Integration Phase (Early 2010s–2020)

With the rapid advancement of multi-gene panels and whole-exome/genome sequencing technologies, research entered the high-throughput era. This phase enabled the simultaneous detection of hundreds of cardiomyopathy-associated genes, significantly improving detection rates while advancing the identification of compound heterozygous mutations and *de novo*mutations (19). The markedly improved mutation detection rate during this phase substantially advanced the refined classification of pediatric cardiomyopathies, providing scientific foundations for risk stratification and genetic counseling. Furthermore, by integrating genomic, imaging, and clinical phenotype data (20), the criteria for assessing the pathogenicity of variants were optimized, offering more precise diagnoses for affected children.

3. Precision Intervention and Dynamic Management Phase (Early 2020s–2024)

The current research frontier exhibits two major characteristics. First, the advancement of molecular diagnostic technologies, manifested in the use of biomarkers such as liquid biopsy (e.g., ctDNA), proteomics, and miRNA for early diagnosis, efficacy evaluation, and recurrence monitoring (21), thereby providing pediatric patients with precision diagnosis and treatment plans. Second, the integration of artificial intelligence and digital technologies, where AI models are employed to decipher complex genetic variations (22), effectively addressing challenges in detecting complex structural variants (e.g., duplications, insertions, deletions, inversions, translocations) and mosaic mutations. Additionally, AI algorithm-based wearable devices equipped with built-in sensors enable real-time monitoring of critical indicators like electrocardiograms and heart rate in diagnosed children (23). These devices offer quantifiable digital health management throughout the entire treatment cycle.

Keyword bursts can be used to analyze research frontiers in a field. The burst word map for pediatric cardiomyopathy genetics research (Figure 7) shows that since 2000, the emergence of research hotspots in genetic testing for pediatric cardiomyopathy has primarily occurred in three periods. In the early stage (2000–2009), the four burst words were “gene,” “deficiency,” “Barth syndrome,” and “expression,” with burst strengths of 15.7, 8.43, 7.7, and 6.65, respectively, and duration times of 14, 14, 13, and 7a (where “a” represents annual units). This indicates that research during this period focused on single-gene defects and specific syndromes, representing preliminary genotype-phenotype association studies. In the middle stage (2010–2020), the five burst words were “protein,” “disorders,” “phenotype,” “disease,” and “task force,” with burst strengths of 8.34, 6.88, 6.91, 7.06, and 7.88, respectively, and duration times of 8, 10, 7, 3, and 4a. During this period, research shifted toward exploring the underlying pathogenesis and developing clinical guidelines, becoming increasingly systematic. In the recent stage (2021–present), the two burst words were “pediatric cardiomyopathy” and “genetics,” with burst strengths of 6.13 and 7.19, respectively, and duration times of 4 and 3a. The sustained prominence of these keywords marks the entry of genetic research in pediatric cardiomyopathy into a phase of in-depth development centered on pediatric population specificity and comprehensive genetic analysis. This phase aligns with the precision management goals of the third research phase in the timeline map.

By synthesizing the timeline map (Figure 6) and burst map (Figure 7), although research directions in pediatric cardiomyopathy genetics are relatively scattered, there remains a dynamic evolutionary process. The first area of focus is applied research in diagnostic testing technologies. The diagnosis of pediatric cardiomyopathy has evolved from initial single-gene screening to multi-gene sequencing, and further to AI-based multi-omics integrated analysis. Advances in detection technologies have significantly improved the detection rate of genetic mutations and the accuracy of clinical phenotype diagnosis, greatly reducing the likelihood of misdiagnosis and subsequent treatment delays for patients.

Secondly, significant progress has been made in precision treatment technologies. The research pathways have evolved from initially exploring the association between clinical phenotypes and genetic characteristics to systematically elucidating the molecular mechanisms of disease onset. Efforts have also been focused on constructing predictive models for conditions such as inherited cardiomyopathies and syndromic cardiomyopathies (24–26), enhancing the specificity and accuracy of treatment for affected children. This advancement is driving the shift in clinical therapeutic strategies from traditional empirical approaches to personalized, genetically informed targeted interventions.

Finally, there is the continuous evolution of prognostic assessment techniques. The prognosis evaluation for pediatric cardiomyopathy has progressed from initial static risk stratification based on single-point data to a dynamic monitoring system integrating imaging and biomarkers. It is further advancing toward an intelligent digital management phase that combines artificial intelligence with real-time physiological data. Correspondingly, the objectives of genetic testing have shifted from a one-time diagnostic confirmation to leveraging digital health platforms for long-term monitoring, proactive risk alerts, and personalized treatment adjustments.

#### 3.4.2 Research topic identification

The BERTopic model was employed for in-depth topic mining, aiming to more accurately analyze the evolutionary characteristics and practical logic of genetic research in pediatric cardiomyopathy (27). This model leverages the pre-trained contextual embeddings to capture the implicit semantic associations of specialized terms such as “genetic diagnosis” and “genetic screening.”

**Figure 8:**
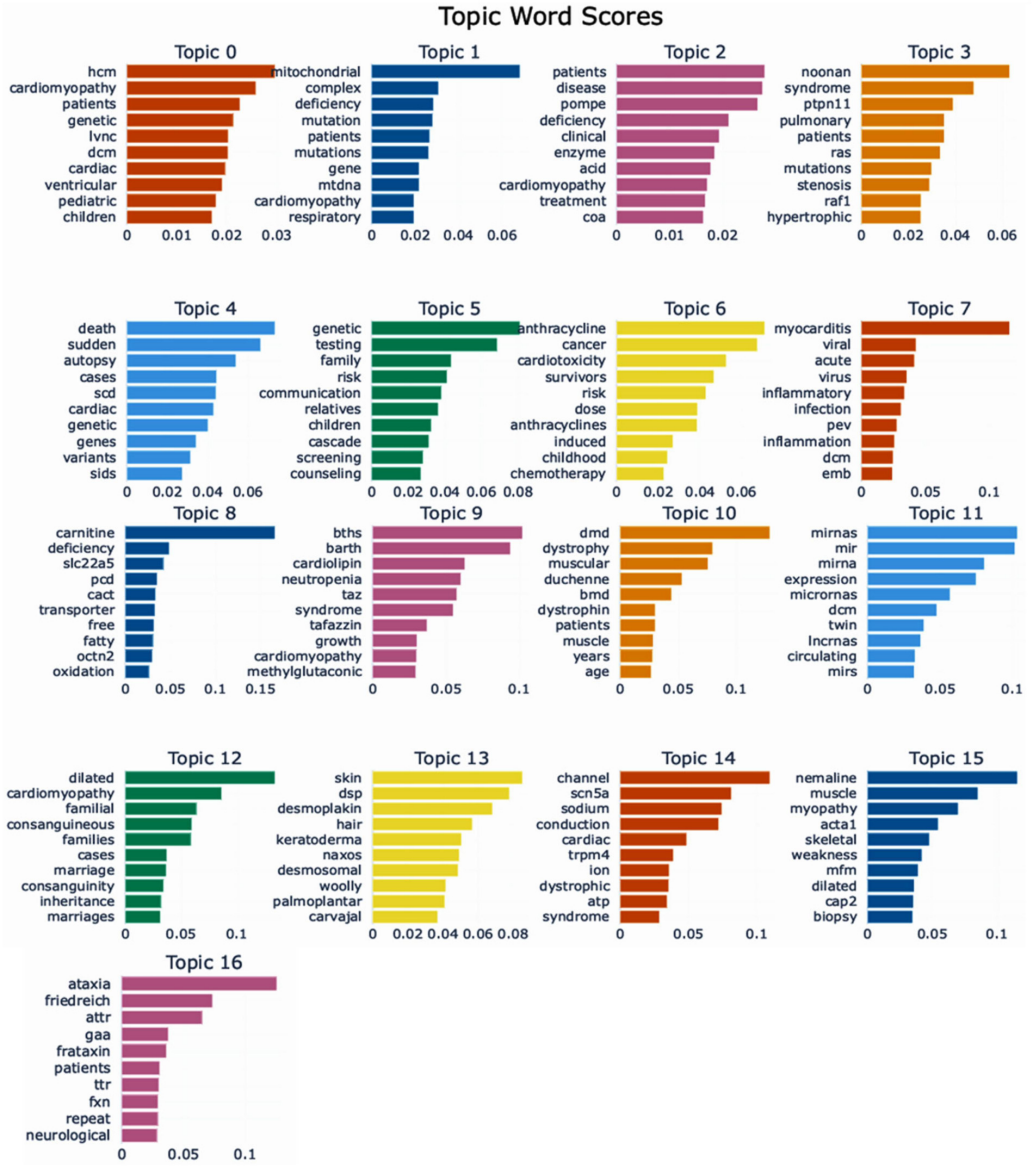
The research topics of pediatric cardiomyopathy genetic.

Following automated iterative processing, the BERTopic model extracted 17 semantically distinct and clinically significant research topic clusters from the research article abstracts (Figure 8). These topics encompassed keywords across multiple dimensions, including the etiology, diagnosis, and treatment of pediatric cardiomyopathy, collectively forming the core knowledge framework for its clinical management. Researchers focused not only on the nature and pathogenesis of the disease but also emphasized prognostic outcomes and preventive strategies. This thematic analysis provides a comprehensive understanding of the knowledge structure and research trends within pediatric cardiomyopathy genetics.

Based on the semantic connotations of the keywords, research content, and clinical logic associated with each topic, this study manually summarized the themes in the field of pediatric cardiomyopathy genetics. According to the semantic focus of the keywords in Figure 8 and their functional positioning in the articles, the 17 topics were further grouped into five core research directions: (1) clinical phenotype classification of pediatric cardiomyopathy, (2) etiology and pathogenesis of pediatric cardiomyopathy, (3) diagnostic techniques for pediatric cardiomyopathy, (4) risk assessment and stratification of pediatric cardiomyopathy, and (5) genetic counseling and ethics in pediatric cardiomyopathy. To clearly illustrate this inductive process, Table 2 presents the mapping relationship between the 17 topic clusters and the five research directions.

**Table 2:** Mapping and cross-associated relationships between BERTopic topics and core research directions.

Topic ID	Primary research direction	Cross-associated research directions	Cross-associated connotation and mapping rationale
Topic 0	Clinical phenotype classification	Diagnostic techniques	Focusing on hereditary cardiomyopathy phenotypes as the core content, while frequently featuring keywords such as genetic mutations and pathogenic sites. This topic serves both as an important component of cardiomyopathy classification and as a primary target for the application of molecular diagnostic techniques.
Topic 1	Etiology and pathogenesis	Clinical phenotype classification, diagnostic techniques	Focusing on pathogenic mechanisms such as mitochondrial metabolic abnormalities, corresponding to metabolic cardiomyopathy phenotypes, while also involving mitochondrial gene mutation testing. This topic possesses triple attributes: mechanistic elucidation, phenotypic classification, and molecular diagnosis.
Topic 2	Etiology and pathogenesis	Clinical phenotype classification	Centered on the pathogenic pathways of metabolic diseases such as Pompe disease, directly corresponding to the clinical classification of metabolic cardiomyopathy. With mechanisms as the core, it also serves to define disease phenotypes.
Topic 3	Clinical phenotype classification	Etiology and pathogenesis, Diagnostic techniques	Corresponding to syndromic cardiomyopathy phenotypes such as Noonan syndrome, while revealing the molecular mechanisms of RAS pathway abnormalities and involving mutation screening of specific pathogenic genes. This topic spans classification, mechanism, and diagnosis.
Topic 4	Etiology and pathogenesis	Risk assessment and stratification	Taking ion channel abnormalities and electrophysiological disturbances as the core pathogenic mechanisms, directly linked to malignant outcomes such as sudden cardiac death. This topic serves as an important link between mechanistic research and sudden death risk stratification.
Topic 5	Risk assessment and stratification	Genetic counseling and ethics	Focusing on family risk assessment and cascade screening as core content, while extending to genetic counseling and ethical issues such as communication of familial genetic information and informed consent.
Topic 6	Etiology and pathogenesis	Risk assessment and stratification	Focusing on acquired etiologies such as anthracycline-induced cardiotoxicity, while also involving risk prediction and stratified management of chemotherapy-related myocardial injury.
Topic 7	Clinical phenotype classification	Etiology and pathogenesis	Taking acquired phenotypes such as myocarditis and inflammatory cardiomyopathy as the main body, while also including keywords related to pathogenic mechanisms such as viral infection and immune inflammation.
Topic 8	Etiology and pathogenesis	Clinical phenotype classification, Diagnostic techniques	Focusing on carnitine deficiency and fatty acid oxidation disorders, this topic pertains to the pathogenesis of metabolic cardiomyopathy, while also corresponding to the classification of metabolic cardiomyopathy and related genetic diagnosis.
Topic 9	Genetic counseling and ethics	Clinical phenotype classification	Focusing on X-linked inherited cardiomyopathies such as Barth syndrome, involving familial inheritance patterns and reproductive risks, with genetic counseling ethics as the core, while also corresponding to specific cardiomyopathy phenotypes.
Topic 10	Clinical phenotype classification	No obvious cross-association	Centered on neuromuscular disease-associated cardiomyopathies such as Duchenne muscular dystrophy (DMD), the keywords in this topic are highly concentrated on disease classification, with relatively weak cross-cutting attributes.
Topic 11	Etiology and pathogenesis	Diagnostic techniques	Focusing on molecular mechanisms such as miRNA regulation, while also utilizing circulating miRNAs as non-invasive biomarkers for early diagnosis, possessing both mechanistic and diagnostic value.
Topic 12	Clinical phenotype classification	Genetic counseling and ethics	Focusing on the phenotype of familial dilated cardiomyopathy as the core, while also involving consanguineous marriage and familial inheritance, linking to genetic risk communication and ethical considerations.
Topic 13	Genetic counseling and ethics	Clinical phenotype classification	Corresponding to desmosome-related cardiomyopathy phenotypes, with emphasis on family counseling and risk disclosure under autosomal recessive inheritance patterns.
Topic 14	Etiology and pathogenesis	Clinical phenotype classification	Focusing on mechanisms of ion channel and sodium channel abnormalities, corresponding to channelopathy-related cardiomyopathy phenotypes, with mechanism and classification highly intertwined.
Topic 15	Clinical phenotype classification	No obvious cross-association	Centered on neuromuscular disease-associated cardiomyopathies such as nemaline myopathy, with the topic content concentrated on phenotypic classification.
Topic 16	Genetic counseling and ethics	Clinical phenotype classification	Corresponding to Friedreich's ataxia-associated cardiomyopathy phenotypes, with emphasis on late-onset disease risk and cross-age ethical issues.

Research direction 1 refers to the classification and clinical phenotypes of pediatric cardiomyopathies, including inherited cardiomyopathies (Topics 0, 7, 12), metabolic cardiomyopathies (Topics 1, 2, 8), acquired cardiomyopathies (Topics 6, 7), and cardiomyopathies associated with neuromuscular disorders (Topics 10, 15). These articles can reflect researchers' in-depth exploration of different cardiomyopathy types and their interrelationships, aiming for more accurate diagnosis and treatment of pediatric cardiomyopathy subtypes, while providing a taxonomic foundation for precision medicine and prognosis assessment.

Research direction 2 focuses on the etiology and pathogenesis. By examining the molecular mechanisms (Topic 11), metabolism and energy homeostasis (Topics 1, 2, 8), as well as ion channels and electrophysiological abnormalities (Topics 3, 4, 14) in pediatric cardiomyopathy, it delves into the biological foundations of disease development. These articles have revealed the complexity of non-single etiologies and explored the pathways from genetic variations to functional abnormalities, contributing to a more comprehensive understanding of the disease's pathogenesis.

Research direction 3 reflects the evolution of diagnostic techniques from single-gene testing to molecular diagnostics. Genome/exome sequencing, which focuses on pathogenic DNA-level mutations (e.g., *MYH7*, *MYBPC3*, etc.), has become a core method for etiological diagnosis, particularly crucial for identifying syndromic, metabolic, and *de novo*mutations (Topics 0, 1, 3, 8). However, a significant proportion of pediatric patients remain undiagnosed by these approaches. Biomarker detection technologies (Topic 11) enable early diagnosis of pediatric cardiomyopathy by identifying differential expression profiles in serum or exosomes. Moreover, different etiologies of cardiomyopathy (e.g., HCM, DCM) may exhibit unique miRNA “fingerprint profiles.” Analyzing miRNA expression patterns facilitates more precise differentiation among diseases with overlapping clinical presentations.

Research direction 4 focuses on the risk stratification of pediatric cardiomyopathy, reflecting the researchers’ emphasis on disease prognosis. This direction explores risk assessment and prognostic stratification for patients and their family members based on genetic information, family history, and clinical characteristics (Topics 4, 5).

Research direction 5 reveals the complex challenges in genetic counseling and ethics associated with pediatric cardiomyopathy testing. The application of genetic testing is inherently intertwined with profound ethical considerations. For example, cardiomyopathy-related genetic testing may uncover information about reproductive risks (the possibility of transmission to offspring) or the risk of developing the disease in adulthood, which extends beyond the scope of traditional pediatric medical decision-making. Consequently, genetic testing for pediatric cardiomyopathy presents complex issues regarding informed consent. Moreover, genetic information possesses unique sensitivity and familial relevance, and test results may inadvertently reveal parental genetic status. Even more concerning is the risk that such information could be misused for insurance discrimination or discrimination in education and employment.

It should be noted that the 17 topic clusters automatically generated by the BERTopic model based on semantic similarity do not strictly belong to a single research category. Most topics exhibit multidimensional cross-cutting characteristics in terms of keyword composition, research connotation, and clinical application. For instance, Topic 5 encompasses both research Direction 4 (risk assessment) and Direction 5 (genetic counseling and ethics), as its keywords include “screening” and “risk” as well as “communication” and “counseling”. Furthermore, diagnostic techniques (Research Direction 3) do not appear as a single topic cluster but rather permeate multiple etiology-related topics as a methodological dimension (e.g., genetic testing content in Topics 0, 1, 3, and 8, and biomarker detection in Topic 11). This precisely reflects the high degree of integration between diagnostic techniques and etiological mechanism research. Therefore, the same topic cluster may simultaneously reflect disease phenotype and classification, reveal pathogenesis, support diagnostic techniques, or relate to risk stratification and ethical issues. This cross-associated connotation reflects the intrinsic interconnections within the field of pediatric cardiomyopathy research. It further validates that the BERTopic clustering results are highly consistent with the inherent logic of the discipline.

More importantly, these 17 topic clusters are not merely academic classifications but also have direct implications for the clinical practice of pediatric cardiomyopathy. Specifically: Topic 0 (hereditary cardiomyopathy phenotypes, with frequent mentions of genetic mutations and pathogenic sites) suggests that targeted genetic testing (e.g., sarcomere gene testing for hypertrophic cardiomyopathy) should be incorporated into routine diagnostic workflows. Topics 1, 2, and 8 (mitochondrial disorders, Pompe disease, carnitine deficiency) alert clinicians to perform metabolic screening (serum acylcarnitine profile, urinary organic acids, enzyme activity assays) when a child presents with unexplained cardiomyopathy accompanied by multi-system involvement. Topic 3 (Noonan syndrome/RASopathy) emphasizes the importance of syndrome-directed genetic testing and follow-up for cardiac defects. Topic 4 (ion channel abnormalities and sudden death) directly guides risk stratification for implantable cardioverter-defibrillator (ICD) placement and family screening. Topic 5 (cascade screening and risk communication) provides a framework for family-centered genetic counseling, covering informed consent, reproductive risks, and discussions of discrimination risk. Topic 6 (anthracycline-induced cardiotoxicity) guides cardioprotective strategies during chemotherapy and long-term monitoring. Topic 11 (miRNA biomarkers), although still at the research stage, holds promise as a non-invasive tool for early diagnosis and disease monitoring. Topic 14 (channelopathies) underscores the necessity of electrocardiographic and electrophysiological evaluation. Finally, Topics 9, 13, and 16 (Barth syndrome, desmosomal cardiomyopathy, Friedreich ataxia) raise unique ethical challenges, such as pre-symptomatic testing in minors and disclosure of reproductive risks. In summary, the integrated clinical practice chain of “phenotype-mechanism-diagnosis-risk-management” presented by these topic clusters directly supports the precision medicine approach to pediatric cardiomyopathy.

**Figure 9:**
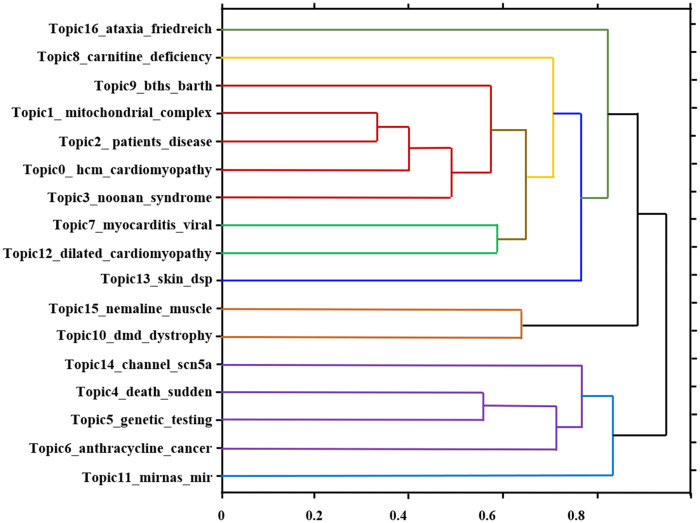
Hierarchical clustering of research topics.

**Figure 10:**
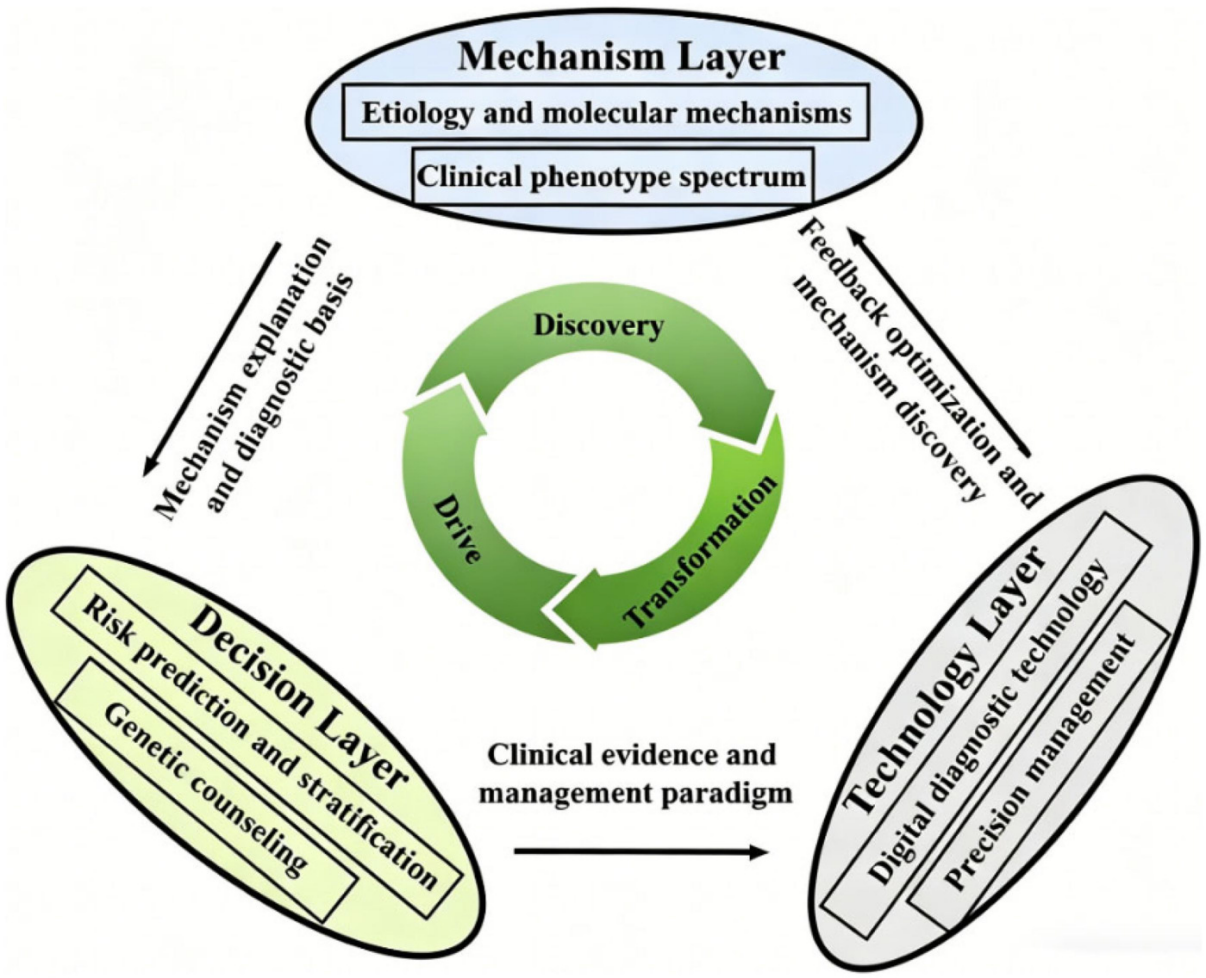
Knowledge structure system of pediatric cardiomyopathy genetics.

To further explore the potential hierarchical structure among the topics, this study employed the built-in hierarchical clustering function of the BERTopic model to visually display the interrelationships between topics (Figure 9). As shown in Figure 10, the hierarchical clustering of topics can be broadly summarized into three logical levels, corresponding to the mechanism layer, the translation/transition layer, and the decision layer. The mechanism layer mainly encompasses etiological and molecular mechanisms (e.g., metabolic pathways, ion channel abnormalities, miRNA regulation) as well as the clinical phenotype spectrum (e.g., hereditary, metabolic, and acquired cardiomyopathies). The translation/transition layer reflects the shift from basic discovery to clinical application, including risk prediction and stratification, genetic counseling, etc. The decision layer focuses on clinical management strategies (e.g., family screening, long-term monitoring). This hierarchical structure reflects the fundamental logic of research content in pediatric cardiomyopathy genetics, progressing from “mechanism discovery” to “clinical translation” and then to “decision application.” It should be noted that this hierarchical classification is a structural observation based on semantic similarity of the topics. It does not represent a strict temporal or developmental sequence.

The keyword timeline (Figure 6) and burst detection (Figure 7) reveal three research stages. These stages are the single-gene targeted screening stage, the high-throughput sequencing and multi-omics integration stage, and the precision intervention and dynamic management stage. It can be observed that the hierarchical clustering results of the BERTopic topics are inherently consistent with the above evolutionary trends. For example, metabolic and ion channel topics within the mechanism layer (e.g., Topics 1, 2, 8, 14) are primarily concentrated in the early and middle articles, whereas topics related to risk stratification and genetic counseling in the decision layer (e.g., Topics 4, 5, 9, 13, 16) are more prominent in recent articles. This correspondence suggests that the topic hierarchical clustering, to a certain extent, corroborates the research trajectory from “basic mechanism exploration” toward “clinical integration and application” described by the bibliometric analysis. Meanwhile, semantic associations exist between some mechanism-related and decision-related topics (e.g., Topics 0, 3, and 11 possess both mechanistic and diagnostic attributes). This indicates possible interactions between mechanism discovery and clinical translation. However, it should be noted that such interactive pathways have specific strength and direction. These remain to be validated by more refined bibliometric methods in future studies.

## 4 Discussion

To reveal the characteristics and development trends of genetic studies in pediatric cardiomyopathy, the current situation and future directions have been summarized. This provides a reference for the future diagnosis and clinical translation of pediatric cardiomyopathy.

### 4.1 Current research status and existing challenges

Over the past 25 years, significant progress has been made in genetic research on pediatric cardiomyopathy, with the field transitioning from the “diagnostic era” to the “precision treatment era.” However, numerous challenges persist in both clinical practice and scientific research, including high genetic heterogeneity, complex genotype-phenotype correlations, and difficulties in clinical translation.

#### 4.1.1 High genetic heterogeneity and challenges in determining pathogenicity

Before the era of genetic testing and big data, a rare variant showing co-segregation in an affected family was often directly determined as pathogenic. With the establishment of large-scale healthy population reference databases such as gnomAD and 1000 Genomes, many variants previously classified as “pathogenic” based solely on familial co-segregation have been reclassified as variants of uncertain significance (VUS), representing a core shift in clinical genetic interpretation. A considerable number of rare variants once considered pathogenic are found to exist at appreciable frequencies in healthy individuals. For example, the *Δ*25 bp deletion in the *MYBPC3* gene has a carrier frequency as high as 4%–5% in the South Asian general population, directly challenging the validity of determining pathogenicity based purely on familial co-segregation and leading to its reclassification as VUS (28). Similarly, *TTN* truncating variants were once classified as pathogenic in cardiomyopathy families. However, after frequency adjustment using gnomAD, a large population database, many low-frequency truncating variants were downgraded from “pathogenic” to VUS. The reasons were relatively high carrier rates in the general population and incomplete penetrance. This widespread phenomenon of variant reclassification suggests that early studies relying on familial co-segregation and predictive models had a high rate of misjudgment (29, 30).

The widespread adoption of high-throughput sequencing enables the simultaneous screening of hundreds of cardiomyopathy-related genes, significantly increasing the number of detected variants. At the same time, this adoption leads to a sharp rise in the proportion of VUS. Most variants detected in pediatric cardiomyopathy patients are novel, rare, or functionally unannotated. Due to a lack of population frequency data, family co-segregation evidence, and functional support, the variant does not meet ACMG/AMP criteria for pathogenicity and can only be provisionally classified as VUS (31). These challenges stemming from technological advances are especially striking in pediatric populations. In children with cardiomyopathy, age-dependent incomplete penetrance is common. For instance, a child carrying *LMNA* or *FLNC* variants may show no obvious hypertrophy or cardiac dysfunction, only developing symptoms later in adulthood. Meanwhile, *de novo*mutations account for a much higher proportion of cases in children than in adults. Adding to this is the lack of pediatric-specific, multi-ethnic local reference databases. As a result, clinical interpretation, follow-up, and the dynamic reclassification of VUS become far more complex.

VUS is not a static or fixed classification. Instead, it represents an intermediate status that can be clarified and reclassified over time as evidence accumulates from population frequencies, family segregation, functional studies, and other sources. Reclassification of VUS mainly relies on three approaches: frequency adjustment using large genomic databases, extended family co-segregation analysis, and molecular functional assays.

Large population databases like gnomAD, ExAC, and the East Asian-focused GenomeAsia 100K provide baseline allele frequencies across different geographic and ethnic groups. This helps correct for background genetic polymorphisms. For example, a *MYH7* missense variant specific to Southeast Asian populations may be rare in European reference databases but present at a low carrier rate in local healthy individuals. Comparing frequency data by population can classify it as a benign polymorphism, allowing downgrading of a previous VUS (32–34). Many truncating variants in the *TTN* gene have low-penetrance background carriage in the general population. Using gnomAD's large-scale population frequency data, variants previously misclassified as pathogenic can be revised to benign or likely benign (35).

Extended family co-segregation analysis (36) follows probands along with their parents, siblings, and multiple generations to test co-segregation of clinical phenotypes and genetic variants. This effectively validates the genetic association between a variant and disease. Take a missense or deletion variant in *MYBPC3* from a family with hypertrophic cardiomyopathy. Initially, it may only be classified as VUS, insufficient to determine pathogenicity. But if extended family follow-up shows the variant co-segregating with phenotypes like hypertrophy and ventricular remodeling across generations, and the pattern matches known autosomal dominant inheritance, then enough co-segregation and clinical evidence can upgrade the VUS to likely pathogenic or even pathogenic (37).

Functional assays use *in vitro* cell models, cardiac organoids, protein expression and enzyme activity tests, immunofluorescence localization, and other methods to show pathogenic effects at the molecular level. For instance, a variant in *DES* (encoding desmin) can disrupt intermediate filament assembly, lead to abnormal protein aggregation inside cells, and eventually worsen cardiomyocyte apoptosis. Functional evidence from cell and animal models provides key support under ACMG/AMP PS3 criteria, upgrading such VUS to pathogenic (38). Similarly, truncating and missense VUS in *FLNC* can be characterized through cardiomyocyte contraction assays and myofibril structure observation to determine pathogenic potential and support reclassification (39).

As ACMG/AMP guidelines for variant interpretation are widely adopted, non-European multi-ethnic genomic databases continue to improve, functional testing systems for cardiomyopathy become more standardized, and long-term clinical follow-up data from pediatric cardiomyopathy cohorts accumulate, some VUS related to childhood cardiomyopathy can be more precisely reclassified as benign, likely benign, likely pathogenic, or pathogenic. This has improved the timeliness and accuracy of genetic interpretation to some extent.

Still, constraints exist. Limited access to functional validation, incomplete family structures, and complex inheritance patterns mean many VUS will remain unclassified for a long time. Progress in this area will require continued technical improvements and data accumulation.

In pediatric cardiomyopathy clinical practice, VUS are common and their dynamic reclassification has real, practical impacts on disease diagnosis, long-term risk stratification, family genetic counseling, and individualized treatment decisions.

At the diagnostic level, genetic testing yielding only VUS cannot serve as a definitive basis for the diagnosis of hereditary cardiomyopathy, making it difficult to establish a clear genotype–phenotype correlation. For example, a child with unexplained left ventricular hypertrophy or a restrictive cardiomyopathy phenotype, but only a *FLNC* VUS, lacks sufficient evidence for pathogenicity. The usual approach is long-term clinical follow-up and serial imaging monitoring. In most cases, it is difficult to directly attribute the myocardial disease to that genetic variant (40). Similarly, when a child with dilated cardiomyopathy carries a VUS in genes like *SGCD* or *SCN5A*, that alone cannot confirm hereditary cardiomyopathy. Clinically, acquired causes such as viral myocarditis or nutritional-metabolic disorders must still be ruled out.

At the prognostic evaluation level, children carrying a VUS cannot refer to risk thresholds established for clearly pathogenic variants. This makes it hard to precisely stratify risks for sudden death, progressive heart failure, or malignant arrhythmias. Evidence from international multicenter cohort studies (mainly in adults) shows that carriers of clearly pathogenic *LMNA* variants (especially truncating ones) are high-risk and need intensified Holter monitoring, cardiac MRI, and early-to-mid intervention. For a child with only an *LMNA* VUS, however, existing evidence provides no uniform risk stratification standard. Therefore, standardized, individualized follow-up intensity and warning protocols are not yet available. This management dilemma highlights the need for more functional validation and case follow-up for such genes (41).

At the genetic counseling level, the uncertainty of VUS poses great challenges to parental reproductive reassessment as well as screening of siblings and family relatives, rendering precise estimation of disease recurrence risk infeasible. For instance, when a hypertrophic cardiomyopathy family has a *MYBPC3* VUS, genetic counselors cannot reliably estimate the inheritance probability for offspring, nor can they standardize screening or health management for family carriers. A systematic review of 248 VUS from a dedicated cardiomyopathy clinic (42) found that 95.2% remained uncertain long-term, and only 1.6% were upgraded to pathogenic. Most unclassified VUS could not undergo co-segregation or functional validation due to lack of family data. This thoroughly illustrates the real depth of the VUS dilemma in genetic counseling.

At the therapeutic decision-making level, clinicians cannot use a VUS result to guide genotype-directed targeted therapy, exercise restrictions, or precision pharmacotherapy. Management remains empirical symptomatic treatment, such as standard heart failure or antiarrhythmic drugs. Children with clearly pathogenic sarcomere gene variants can be strictly restricted from high-intensity competitive sports and started early on myocardial protection. In contrast, VUS carriers have no clear diagnostic or treatment guidelines and typically receive conservative follow-up and general lifestyle advice.

In the future, we should build regional multicenter prospective cohorts specifically for pediatric cardiomyopathy, establish standardized functional verification platforms for cardiomyopathy-related gene variants, and adopt deep learning and AI models to predict variant pathogenicity. We also need to implement an annual regular review and batch reanalysis system for variants detected in clinical samples. By accumulating evidence on population frequency, familial cosegregation and functional experiments, we can gradually clarify the pathogenicity of VUS. This will lower the clinical detection proportion of VUS, and improve the practicality, etiological interpretation ability, and individualized clinical guidance of genetic testing for pediatric cardiomyopathy. Notably, the ClinGen Cardiomyopathy Variant Curation Expert Panel (VCEP) updated the ACMG/AMP criteria for eight sarcomeric genes including *MYH7* and *MYBPC3* in 2024. The revision raised the reclassification rate of partial VUS to 17.4%, providing a practical reference for standardizing the genetic diagnosis of cardiomyopathy (43).

Current genetic testing technologies also have significant limitations. Among pediatric cardiomyopathy patients with existing clinical phenotypes, only 30% to 60% can be identified clear pathogenic mutations through existing genetic testing methods (40). This limitation stems from the marked genetic heterogeneity of pediatric cardiomyopathy-not only are there numerous pathogenic genes [such as sarcomere protein genes *MYH7* and *MYBPC3* (44), cytoskeletal protein genes *DMD* and *SGCD* (45), and mitochondrial-related protein genes (46, 47)], but the inheritance patterns are also diverse (e.g., autosomal dominant/recessive, X-linked, mitochondrial inheritance, etc.) (2, 48). The clinical manifestations are highly variable. Sanger sequencing and conventional whole exome sequencing don't fully cover non-coding regulatory regions. They also have limited power to detect large structural variants, mosaicism, or deep intronic splice variants. Nor can they directly assess whether epigenetic abnormalities like DNA methylation play a pathogenic role in pediatric cardiomyopathy. As a result, many children with clear clinical phenotypes still lack a definitive genetic diagnosis.

#### 4.1.2 Racial and age bias in genetic databases

Current mainstream gene databases (e.g., gnomAD, ClinVar, HGMD) rely heavily on genomic data from European and American populations, with adult cases constituting the overwhelming majority, while data on Asian children remains insufficient (49, 50). This structural bias creates a dual dilemma for the precise diagnosis of cardiomyopathy in Asian children. First, racial differences mean that many variants rare in European and American populations may represent genetic polymorphisms in Asian populations. Directly applying existing annotations can easily lead to “over-interpretation” and misdiagnosis. Second, the critical gap in age representation is particularly problematic. Pediatric cardiomyopathies, especially those manifesting in infancy, often involve de novo mutations or severe metabolic-related genes rarely seen in adults. The lack of database correlations between pediatric-specific allele frequencies and phenotypes may trap pathogenicity assessments in cognitive biases of overgeneralization. Additionally, databases inadequately incorporate information from healthy carriers across age groups, leading to certain variants with relatively high carrier rates in the general population being erroneously labeled as “pathogenic.” Clinically, however, corresponding phenotypes remain elusive. Ultimately, this may impose unnecessary psychological burdens on affected children and their families while prompting misguided clinical interventions.

From the perspective of public health, broader systemic challenges can arise from data bias. Firstly, the rational allocation and equity of public resources in precision medicine have been directly affected. Diagnostic guidelines and variant interpretation standards based on biased data may exhibit reduced efficacy in non-European/American pediatric populations, leading to public health investments (such as genetic screening programs and access to targeted therapies) failing to achieve expected health outcomes, thereby exacerbating existing global health inequities. Secondly, the scientific formulation of population-level disease prevention and early screening strategies has been seriously restricted. In the absence of population-specific baseline data, public health authorities struggle to accurately determine the true “background noise” of pediatric cardiomyopathy-associated genetic variants in the general population, making it impossible to scientifically establish thresholds and scopes for neonatal or childhood genetic screening. Direct use of non-native data may raise false-positive rates in screening, waste medical resources, increase family anxiety, and overload follow-up systems. It may also miss region-specific high-risk variants and delay early intervention.

#### 4.1.3 Lack of genotype-phenotype association models

Despite considerable efforts by researchers to establish models linking genetic variants to clinical phenotypes in pediatric cardiomyopathy, no breakthrough progress has been achieved to date (51, 52), constituting one of the primary obstacles to realizing precision medicine. The challenges are exceptionally complex. First, the phenotypes of pediatric cardiomyopathy exhibit significant heterogeneity and variability. Within different individuals and even within the same family, identical pathogenic mutations in the same gene (e.g., *MYH7* or *TNNT2*) may trigger entirely distinct cardiomyopathy types, such as HCM, DCM, or even RCM, with widely varying disease severity, age of onset, and progression rates (53). Second, the complex association is heavily influenced by multiple factors. Beyond the primary gene mutation, an individual's genetic background (e.g., modifier genes), intrinsic physiological environment (e.g., age, sex), and extrinsic environmental factors collectively form an intricate regulatory network. More importantly, epigenetic regulation (e.g., DNA methylation, histone modifications), as a critical bridge linking genes and the environment, can significantly influence gene expression without altering the DNA sequence, further increasing the uncertainty of phenotype prediction (54). Consequently, the predictive accuracy of existing association models based on single genes or limited clinical parameters remains generally low, rendering them unreliable for individualized long-term prognosis assessment and risk stratification in clinical practice. This highlights the urgent need for more in-depth and integrative research.

#### 4.1.4 Psychological and social impact of genetic testing results on asymptomatic individuals

The psychological and social repercussions following the detection of pathogenic mutations in children with cardiomyopathy are profound and complex. Psychologically, some families may become trapped in a persistent state of “genetic anxiety” (55–57). Although these children exhibit no clinical symptoms, they may be considered in a “preclinical” stage of the disease, leading their parents or guardians to live in prolonged fear of sudden disease onset. This pervasive stress significantly diminishes the overall quality of life and well-being of the family. Older children, upon learning their genetic status, may experience confusion in self-identity and even develop behavioral issues such as social avoidance. On a societal level, genetic diagnosis carries the real risk of “genetic discrimination” (58, 59). For instance, children may face unnecessary restrictions when enrolling in school or participating in certain sports activities. Their future access to social benefits like commercial health insurance and employment may also be potentially affected. However, the current healthcare system remains inadequately equipped to address such issues, generally lacking specialized psychological counseling and long-term follow-up support systems for asymptomatic child carriers and their families. Additionally, there is no authoritative guideline to standardize information disclosure, safeguard individual rights, or prevent societal discrimination, leaving families and clinicians to navigate numerous uncertainties and ethical dilemmas in practice.

### 4.2 Development trends

#### 4.2.1 AI-driven precision analysis of multi-omics data

Single-genome data cannot fully explain disease heterogeneity. Thus, research has shifted to integrating multi-omics data (genomics, transcriptomics, proteomics, metabolomics) and using AI for deep analysis. In this process, AI plays a central role, primarily in two key aspects.

First, at the genetic variant level, deep learning models [such as REVEL (60) and AlphaMissense (61)] analyze vast amounts of protein sequences and variant data to enable more accurate assessment of the pathogenic potential of missense mutations, improving the interpretation of rare variants and variants of uncertain significance. Second, at the phenotypic level, AI technologies can automate the analysis of complex medical data, such as cardiac MRI, ultrasound imaging, and dynamic electrocardiograms, precisely quantifying subtle structural changes and functional abnormalities that are difficult to detect with traditional visual observation (62, 63). AI-based “digital biomarker” mining not only enhances diagnostic objectivity but also identifies subtle pathological changes in early-stage disease that are often missed by conventional examinations.

Currently, AI has become an important tool for data analysis, risk prediction, and prognosis assessment. AI models trained on multi-omics and clinical data can provide individualized predictions for disease progression risk, heart failure development, malignant arrhythmias, and sudden death risk in pediatric patients. They can also anticipate genotype-phenotype association trends in advance, offering prospective evidence for early clinical intervention, genetic counseling, and family cascade screening. Clinical practice has shown that AI models trained on electrocardiograms and cardiac imaging can achieve automatic identification of cardiomyopathy subtypes, with a diagnostic accuracy of 96% (64, 65). These models also increase screening efficiency by three-fold and reduce the misdiagnosis rate to below 2%. In real-world data covering more than 40,000 scans, an AI system successfully identified 112 previously undiagnosed cases of hypertrophic cardiomyopathy, significantly improving the early detection rate (66). Through AI-driven integrative analysis of multi-omics data, research is gradually uncovering the early changes of pediatric cardiomyopathy at both the molecular level and the tissue structural level. This provides new possibilities for a deeper understanding of disease pathogenesis and for achieving earlier and more precise clinical intervention.

Although AI-driven multi-omics analysis holds great promise for precision diagnosis and treatment of pediatric cardiomyopathy, several major limitations and practical challenges still stand in the way of clinical adoption. These need systematic solutions.

First, data privacy and security. Children are a vulnerable group, so ethical risks are especially high. Multi-center, cross-institutional data integration involves protecting personal and genomic information of pediatric patients, but current compliance-sharing mechanisms remain imperfect. Gaps in data transfer can lead to privacy breaches. Data show that over half of healthcare data security incidents come from poor internal management or staff errors (67). In 2022, the 23andMe data breach affected more than 7 million users. After the company filed for bankruptcy in 2025, genetic data of about 15 million users faced potential sale, highlighting the fragility of genetic data protection in commercial settings (68). The ethical risks for children's genetic data are even more serious. A 2024 investigation revealed that the Greek government secretly launched a newborn whole-genome screening plan, intending to collect DNA from 100,000 newborns and grant exclusive ownership to a private company, with no clear informed consent or privacy protection (69). These real examples show that AI-driven multi-omics integration urgently needs a full-lifecycle security system covering data collection, transmission, storage, and secondary use, with stricter ethical rules and regulatory standards specifically for pediatric populations.

Second, lack of algorithm explainability (the “black box” problem). This limits wide use of AI in pediatric cardiovascular clinical decisions. Most deep learning models lack transparency in their decision process, making it hard for clinicians to trace or understand the reasoning, creating a typical black-box dilemma. A recent review clearly states that applying machine learning in precision medicine faces multiple challenges including data privacy frameworks, cybersecurity risks, ethical concerns, and integration into clinical workflows. Opaque algorithms are a core obstacle (70). In pediatric cardiomyopathy multi-omics prediction, a model may output risk or prognosis results but cannot explain the connection between a specific genetic variant, clinical phenotype, and outcome. This fails clinical, ethical, and communication needs (71). Poor explainability further reduces clinical acceptance: some models can be partly explained in adult cohorts, but with pediatric rare variants or low-penetrance mutations they become almost untraceable, so clinicians find it hard to trust or use AI outputs. A systematic review of 203 public medical imaging datasets found that 33% lacked age metadata. Among those with age information, children made up less than 2%, far below their actual clinical proportion. When an AI model trained mainly on adult data is directly applied to children, significant age-related bias appears. For tasks like cardiac enlargement detection, the false positive rate is highest in infants under 2 years old, and the risk of misdiagnosis rises sharply as age decreases. This shows adult models cannot adapt to pediatric developmental anatomy and physiology, and direct transfer clearly carries safety risks (72). Together, these studies show that the black-box nature of AI models not only blocks clinical translation but, without pediatric-specific training data and fairness constraints, may also amplify structural biases already present in clinical practice.

Third, implementation barriers across different healthcare settings. AI models demand high computing power, high-quality labeled data, standardized datasets, and specialized technical staff. In regions with limited equipment, funding, and personnel, adoption is difficult, worsening healthcare disparities (73). Pediatric data volumes are small, labeling is expensive, and standards across centers are inconsistent, limiting model generalizability (74). Some primary care facilities lack genomic testing or data integration capabilities, further restricting deployment. Moreover, most existing models are trained on European-descendant adult data, so they show ethnic bias when used in non-European children, reducing predictive accuracy and harming fairness and applicability.

Future directions. We need continued progress in algorithm transparency, secure data sharing, lightweight model deployment, and pediatric-specific standardized datasets. For algorithm transparency, adopt human-centered approaches that shift evaluation of explainable AI from technical self-checks to clinically unified standards and real-world clinician-patient co-design (75). For secure data sharing, promote cross-state regulatory consortia and data governance bodies, introduce de-identification protections for pediatric data, and adopt re-consent mechanisms for children to create a trustworthy closed-loop data system. For resource-limited settings, develop lightweight, localized AI models combined with edge devices to reduce computing costs and overcome network latency, pushing AI technology into the full process of pediatric cardiomyopathy diagnosis and treatment.

#### 4.2.2 Clinical translation of gene therapy for pediatric cardiomyopathy

Gene therapy strategies for pediatric cardiomyopathy are at a critical juncture transitioning from proof-of-concept to clinical application (76, 77). This progress primarily stems from two major technological breakthroughs: First, the optimization of adeno-associated virus (AAV) vector technology (78), particularly the AAV9 serotype (79), which exhibits high cardiac tropism and has become an efficient vehicle for delivering therapeutic genes or gene-editing tools. This has enabled precise regulation of specific pathogenic genes in preclinical models. Second, the deep integration of CRISPR-Cas9 gene-editing technology with patient-derived induced pluripotent stem cell (iPSC) models (80). This not only provides a personalized platform for validating gene therapy strategies *in vitro*but also advances the development of gene-corrected cell replacement therapies.

Recent clinical data indicate that gene therapy has entered a substantive stage of clinical translation. For example, TN-201, an AAV9-mediated gene therapy for *MYBPC3*-mutant hypertrophic cardiomyopathy, has been supplied in clinical studies (81). A single administration significantly improves myocardial structure and function, demonstrating the potential to reverse the disease phenotype. In infantile Pompe disease, AAV gene therapy (phase I/II clinical trial) has shown that all 12 treated patients were able to discontinue long-term enzyme replacement therapy (ERT), with marked improvement in motor function and maintenance of normal cardiac function (82). Follow-up data for RP-A501, a gene therapy for Danon disease, indicate that 5 out of 6 pediatric patients achieved a ≥ 10% reduction in left ventricular mass index from baseline, a > 80% reduction in cardiac troponin I, and significant improvements in cardiac function and quality of life scores (83). These patients also demonstrated stable disease status and a favorable safety profile during long-term follow-up. Furthermore, mRNA and LNP delivery technologies have achieved breakthroughs in preclinical studies, where a single administration reversed myocardial fibrosis, restored ventricular function, and prolonged survival. This provides a new avenue for non-viral vector-based gene therapy. These advances offer substantial hope for the treatment of pediatric cardiomyopathy.

#### 4.2.3 Wearable-based dynamic digital health monitoring technology

The long-term monitoring and dynamic management of pediatric cardiomyopathy have been evolving towards continuous and refined approaches, leveraging wearable devices and digital health technologies. This advancement is primarily supported by two key technological pillars. First, the increasing maturity of high-sensitivity, low-burden wearable sensor technologies-such as patch-based electrocardiogram (ECG) monitors and photoplethysmography devices-is evident. This maturity enables long-term, non-invasive continuous collection of physiological parameters, including electrocardiographic signals, heart rate variability, and blood oxygen saturation (23, 84, 85). Second, the integration of edge computing with cloud-based AI analytics platforms enables the preliminary processing of massive real-time physiological data locally. The data is then uploaded to the cloud for in-depth integration and intelligent early warning (86).

Multiple clinical studies have confirmed that wearable electrocardiographic monitoring technology has clear clinical value in the risk management of pediatric cardiomyopathy. In the context of arrhythmia screening in children, smart wearable devices can detect 28% of previously undiagnosed arrhythmias, with an outstanding identification capability for malignant arrhythmic events such as supraventricular tachycardia and ventricular fibrillation (87). This demonstrates a significantly better monitoring performance than conventional short-term electrocardiographic monitoring. A study conducted by the Yale University team showed that an AI model based on wearable single-lead electrocardiographic signals achieved excellent screening performance for structural heart disease, with an AUC of approximately 0.88, a sensitivity of 86%, and a negative predictive value of 99% (88). This makes it an efficient, non-invasive, early auxiliary screening tool for structural heart diseases including pediatric cardiomyopathy. The subcutaneous implantable cardiac monitor (Reveal LINQ) enables continuous electrocardiographic recording for up to three years and has significant value in etiological diagnosis of unexplained syncope and concealed arrhythmias in children. Multiple pediatric real-world studies have reported an overall diagnostic yield of 60%–70%, significantly improving the early detection rate of cardiogenic causes and malignant arrhythmias (89). This provides accurate, long-term risk warning for high-risk pediatric patients such as those with hypertrophic cardiomyopathy. The extravascular implantable cardioverter-defibrillator (EV-ICD) has shown promising prospects for the prevention of sudden death in high-risk children. Intraoperative induction testing has confirmed 100% sensitivity for arrhythmia recognition, accurately identifying and effectively terminating malignant arrhythmias such as ventricular tachycardia and ventricular fibrillation, with preliminary validation of both safety and clinical efficacy (90).

Within this framework, the dynamic monitoring system demonstrates multi-layered application value. At the individual level, the system can achieve early warnings for disease fluctuations and potential risks by identifying abnormal ECG segments, detecting arrhythmia events, and analyzing heart rate trends. At the group management level, continuously collected data provides a structured information source for real-world research, aiding in the construction of more precise disease progression models and stratified management strategies. Additionally, through integration with electronic health records and genomic data, pediatric cardiomyopathy management has shifted from “intermittent outpatient follow-ups” to a model of “continuous, real-time monitoring.” This transformation offers timely, objective evidence for early intervention, medication adjustments, and efficacy evaluation, thereby enhancing treatment adherence and improving long-term outcomes.

#### 4.2.4 Establishment of precision genomic data system for pediatric cardiomyopathy

Precision genetic diagnosis of pediatric cardiomyopathy depends on multi-center collaboration and local data systems. Three key directions are needed: ethnic fairness, age-specific accuracy, and standardized interpretation. Building a precise genomic data system for pediatric cardiomyopathy has two main goals. First, correct current structural biases in public genomic databases—too much data from European adults, too little from non-European children. Second, create a child-specific data ecosystem that accurately supports variant interpretation, phenotype association, risk stratification, and clinical decisions.

Real-world studies show clear value from pediatric, multi-ethnic, phenotype-linked genomic data. The North American Pediatric Cardiomyopathy Registry (PCMR) has followed a large pediatric cohort across 98 centers for 30 years. It provides critical support for genotype-phenotype analysis, prognosis, and variant validation (91). Work from this cohort also confirms that regular reinterpretation of genetic test results is clinically important for all types of cardiomyopathy, improving diagnostic accuracy and correcting past errors (92). A Chinese multi-center cohort of pediatric restrictive cardiomyopathy revealed an Asian-specific genetic profile dominated by TNNI3 mutations, highlighting the need for a local pediatric genomic database (93). Under this framework, five core paths will drive development of a precise genomic data system.

First, build multi-ethnic, stratified pediatric cardiomyopathy cohorts. Use regional networks such as the Asia-Pacific Pediatric Cardiac Society (APPCS), the African Society of Pediatric and Congenital Heart Surgery (ASPCHS), and the Association for European Paediatric and Congenital Cardiology (AEPC). Sample by age group (newborns, infants, school-age children) across multiple centers. Systematically collect genomic and clinical phenotype data, focusing on variants from non-European populations.

Second, accelerate development of child-specific genomic variant databases. Examples include the global Pediatric Cardiomyopathy Genomics Consortium (PCGC) database, the NIH Kids First pediatric genomic platform, and China's emerging cardiovascular genetic database for children. These move away from the adult database framework. They organize variant frequencies and longitudinal follow-up data by cardiomyopathy subtype, age group, and ethnicity, and include control data from healthy children. This provides a local reference for interpreting pediatric cardiomyopathy variants.

Third, build international collaborative frameworks for pediatric cardiomyopathy genomics. Cooperation among APPCS, the American Heart Association (AHA), and AEPC is growing. Multi-center, multi-country data can now be pooled across continents and ethnic groups. The EU-funded Solve-RD project is a model for standardized rare disease data sharing across Europe. It combined WES/WGS and phenotype data from 51 centers in 15 countries, re-analyzed over 6,000 rare disease families, and established standardized phenotype collection (HPO terms), ethical data sharing (FAIR principles), and genomic quality control. Its infrastructure offers a mature template for a global multi-center pediatric cardiomyopathy consortium (94).

Fourth, standardize cross-country data sharing and ethical rules. Current ACMG/AMP variant interpretation guidelines are adding pediatric-specific rules, adjusting evidence weights for *de novo*variants and age-dependent low-penetrance variants. The Human Phenotype Ontology (HPO) continues to expand pediatric clinical terms (95) and is increasingly used in global pediatric cardiomyopathy collaborations, aligning phenotype descriptions across centers. International bodies like ISO and GA4GH can develop ethical guidelines specifically for pediatric genomic data sharing, covering de-identification, guardian consent, and privacy protection.

Fifth, integrate real-world clinical registries with genomic big data platforms. Connect existing national pediatric cardiomyopathy registries to genomic platforms. This allows dynamic linking of clinical phenotypes (e.g., hypertrophy severity, heart failure progression, transplant outcomes) with genetic variants, providing real-world evidence for VUS validation and reclassification. The UK NIHR Biomedical Research Centre has built a inherited cardiac disease database combining sequence and phenotype data from over 8,000 patients and families to aid variant interpretation and family screening (96). North America's PCMR long-term follow-up has confirmed the value of deep integration between real-world registries and genomic platforms for dynamic VUS reclassification (92).

On this basis, AI algorithms and local models can further improve prediction. Use transfer learning and graph neural networks to combine established genomic data with small local pediatric samples, building variant pathogenicity prediction models for non-European children. Also use local ancestry inference (LAI) and population-specific frequency correction to improve allele frequency estimation, making genetic interpretation fairer and more accurate (97, 98).

### 4.3 Limitations

CiteSpace software and the BERTopic topic modeling method enable visual analysis of large volumes of articles, helping researchers quickly understand the current research status and development trends, and roughly predict future directions. Nevertheless, this study still has several limitations, which should be given serious attention when interpreting the global trends in pediatric cardiomyopathy genetics and precision medicine.

1. This study followed strict inclusion and exclusion criteria to screen the literature step by step. However, there was still some heterogeneity in literature quality and topic relevance. Differences existed in study design, sample size, research perspective, and methodological quality across the field. As a result, the strength of evidence in some studies varied. In addition, a few studies had limited relevance to our focus on genetics and precision medicine. It was difficult to fully exclude cases that did not exactly match the core scope. This may have affected the accuracy and purity of our analysis to some extent.
2. This study only searched English articles in Web of Science and PubMed. It did not include other databases or articles in Chinese, German, Italian, or other languages. This may have caused some literature to be missed. As a result, the analysis of spatial distribution, research hotspots, and emerging trends may not be fully accurate. Using only English articles inevitably introduces language bias. This affects the completeness and representativeness of the findings in several ways.

First, excluding non-English articles may systematically underestimate scientific contributions from non-English-speaking countries and regions. Asia, Latin America, and the Mediterranean region have produced many important results in recent years on pediatric cardiomyopathy. These include clinical phenotypes, gene spectra, population-specific variants, and local diagnostic and treatment strategies (99, 100). However, these studies are not well represented in English databases. This leads to a one-sided view of global research contributions. Some early classic studies on cardiomyopathy genetics from European countries (e.g., Germany, France, Italy) were published in local languages. Missing these milestone studies may affect the accurate understanding of the historical development of the field (101, 102). In addition, some subtypes of pediatric cardiomyopathy that are more common in specific regions (e.g., the *MYH7* and *MYBPC3* mutation spectra in East Asian populations, or tropical cardiomyopathy) are rarely published in English. This can cause a gap between the research hotspots shown by bibliometric analysis and the real-world situation (103, 104).

Second, using only English articles may distort the true picture of international research collaboration. Multilateral or regional collaborative projects led or deeply involved by non-English-speaking countries are more likely to be missed. This makes it impossible for collaboration networks, country linkage strengths, and cluster structures in bibliometric analysis to fully reflect real global collaboration patterns. For example, regional multicenter pediatric cardiomyopathy groups led by China, Japan, Brazil, and other non-English-speaking countries have become important research forces. These include the Asia-Pacific Pediatric Cardiac Association (105), the Japanese Society of Pediatric Cardiology and Cardiac Surgery (106), and the Brazilian CHildren and Adolescent Registry in Myocarditis and Cardiomyopathy (107). These platforms have produced high-quality results on population-specific mutation spectra, family co-segregation data, regional treatment strategies, and prognostic risk models. However, some of these results were published in Chinese, Japanese, or Portuguese. Collaboration network maps based only on English searches cannot fully capture these relationships.

Third, missing non-English articles directly interferes with topic distributions and cluster structures. Region-specific causes, population-specific genotype-phenotype associations, regional guidelines, and localized precision medicine practices are more often published in local languages. Their absence can cause bias in hotspot extraction, topic evolution, and cluster analysis. For example, countries in the Mediterranean region (e.g., Spain, Greece, Arab countries in the Middle East) have unique resources such as consanguineous families and founder mutations. High-quality studies on clinical follow-up, gene co-segregation analysis, and founder mutation identification in familial cardiomyopathy are often published in local languages (108, 109). Only including English articles would systematically miss these valuable family data. This would make the global mutation spectrum, genotype-phenotype features, and VUS interpretation strategies incomplete.

Furthermore, language bias may worsen existing regional imbalances in databases. It further amplifies the tendency to focus on English-based research systems. Differences in disease causes, treatment models, and research hotspots across language regions cannot be accurately described. Some regionally common cardiomyopathy subtypes and local clinical experiences cannot be fully presented. This affects the objective judgment of the global research landscape and development trends.

In summary, the trends summarized in this study are more aligned with the characteristics of English-led research. Caution should be taken when generalizing these findings to a truly global scope. In the future, expanding the range of databases and conducting multilingual literature searches can help reduce language bias. This will improve the comprehensiveness, representativeness, and generalizability of the analysis, and present global research diversity in a more balanced way.

3. There is currently no unified standard for parameter settings and analysis methods in CiteSpace. To some extent, this affects the reproducibility of the results. In terms of data statistics and analysis, this study only conducted in-depth analyses of the most productive countries, institutions, and keywords. The study did not analyze each individual article to fully explore associations among article details, and in the co-citation analysis, only first authors were analyzed. The issue of distinguishing different authors with identical names could not be precisely resolved, which may introduce a certain degree of bias. In addition, some recently published high-quality studies have low citation counts because they have been out for a short time. These studies are not fully reflected in the bibliometric analysis. As a result, the analysis results may not fully match the actual progress in the field.

Despite these limitations, this study systematically reviewed the literature on pediatric cardiomyopathy genetics over the past 25 years. The data are sufficient and the time span is complete. The findings can still objectively reflect the core research hotspots, evolution paths, and overall development trends in this field. They can provide reliable and useful references for future researchers.

Future bibliometric studies could further expand the range of databases. They could include more languages and update collaboration networks with the latest publications. More unified methodological standards would also help. These steps would improve the accuracy, comprehensiveness, and global representativeness of the results.

## 5 Conclusion

This manuscript reviews the global research landscape in pediatric cardiomyopathy genetics over the past 25 years. Data analysis confirms sustained research activity in this field, which has remained a central focus of global pediatric cardiovascular disease studies. Research evolution has progressed from early reliance on single-gene screening to a phase marked by genomic sequencing and multi-omics integration, advancing toward AI-driven precision analysis, dynamic risk stratification, and targeted therapies. However, several key barriers to precision medicine persist: high genetic heterogeneity of genetic variants in pediatric cardiomyopathies, limited population diversity in public databases, and unclear genotype–phenotype correlations. These challenges restrict the development of data-driven clinical management and translational applications. Future research should focus on exploring the functional mechanisms of pathogenic genetic variants, developing AI analysis systems capable of efficiently integrating multi-omics data, and building more population-representative pediatric genetic databases. Concurrently, efforts should focus on integrating dynamic physiological data from digital health monitoring tools, such as wearable devices, with genetic information to collectively advance the diagnosis and treatment of pediatric cardiomyopathy toward a digitalized, precise, and personalized paradigm. During this process, it is essential to build a full-life-cycle system for privacy protection and security compliance that spans data collection, storage, sharing and application. This ensures multi-source data integration proceeds safely under ethical oversight.

## Data Availability

The raw data supporting the conclusions of this article will be made available by the authors, without undue reservation.
